# Ubiquitination of acetyltransferase Gcn5 contributes to fungal virulence in *Fusarium graminearum*

**DOI:** 10.1128/mbio.01499-23

**Published:** 2023-07-28

**Authors:** Ahai Chen, Yifan Zhou, Yiyi Ren, Chao Liu, Xingmin Han, Jing Wang, Zhonghua Ma, Yun Chen

**Affiliations:** 1 State Key Laboratory of Rice Biology and Breeding, Institute of Biotechnology, Zhejiang University, Hangzhou, China; The University of Georgia, Athens, Georgia, USA

**Keywords:** histone acetyltransferase Gcn5, E3 ligase Tom1, deubiquitinating enzyme Ubp14, *Fusarium graminearum*, virulence

## Abstract

The histone acetyltransferase general control non-depressible 5 (Gcn5) plays a critical role in the epigenetic landscape and chromatin modification for regulating a wide variety of biological events. However, the post-translational regulation of Gcn5 itself is poorly understood. Here, we found that Gcn5 was ubiquitinated and deubiquitinated by E3 ligase Tom1 and deubiquitinating enzyme Ubp14, respectively, in the important plant pathogenic fungus *Fusarium graminearum*. Tom1 interacted with Gcn5 in the nucleus and subsequently ubiquitinated Gcn5 mainly at K252 to accelerate protein degradation. Conversely, Ubp14 deubiquitinated Gcn5 and enhanced its stability. In the deletion mutant Δ*ubp14*, protein level of Gcn5 was significantly reduced and resulted in attenuated virulence in the fungus by affecting the mycotoxin production, autophagy process, and the penetration ability. Our findings indicate that Tom1 and Ubp14 show antagonistic functions in the control of the protein stability of Gcn5 via post-translational modification and highlight the importance of Tom1-Gcn5-Ubp14 circuit in the fungal virulence.

**IMPORTANCE:**

Post-translational modification (PTM) enzymes have been reported to be involved in regulating numerous cellular processes. However, the modification of these PTM enzymes themselves is largely unknown. In this study, we found that the E3 ligase Tom1 and deubiquitinating enzyme Ubp14 contributed to the regulation of ubiquitination and deubiquitination of acetyltransferase Gcn5, respectively, in *Fusarium graminearum*, the causal agent of Fusarium head blight of cereals. Our findings provide deep insights into the modification of acetyltransferase Gcn5 and its dynamic regulation via ubiquitination and deubiquitination. To our knowledge, this work is the most comprehensive analysis of a regulatory network of ubiquitination that impinges on acetyltransferase in filamentous pathogens. Moreover, our findings are important because we present the novel roles of the Tom1-Gcn5-Ubp14 circuit in fungal virulence, providing novel possibilities and targets to control fungal diseases.

## INTRODUCTION

Cellular processes are altered via changes in protein abundance or activity in response to endogenous development cues or environmental stimuli. Post-translational modifications (PTMs) are able to regulate protein abundances and activities by adding or removing chemical moieties or modifying groups to or from target proteins. More than 200 different types of modifications have been identified (1, 2). Among them, ubiquitination is a key and ubiquitous PTM in which a 76-amino-acid-long protein (8.5 kDa), i.e., ubiquitin (Ub), is covalently attached to a protein substrate, modulating protein stability, localization, and function of the target proteins (3, 4). Target proteins are ubiquitinated via a series of enzymatic reactions mediated by E1 (Ub-activating enzyme), E2 (Ub-conjugating enzyme), and E3 (Ub ligase). The first step is the activation of inactive Ub by linking the Ub with E1 via a thioester linkage, which depends on ATP hydrolysis. After activation, the Ub molecule is transferred from E1 to an active cysteine residue on E2, forming an E2-Ub intermediate. Finally, a specific E3 recruits the targeted substrate protein and catalyzes the transfer of the active Ub from E2 to the substrate protein by forming an isopeptide bond between the carboxy-terminal glycine of ubiquitin and the target protein, typically at a lysine residue (5 - 7). Since they specifically recognize substrate proteins, E3 ligases are considered as the most key components of the ubiquitin system (8). E3 ligases are commonly classified into three classes: the really interesting new gene (RING) family, the homology to E6AP C-terminus (HECT) family, and the RING-between-RING (RBR) family. Tom1 (temperature-dependent organization in mitotic nucleus 1) is a HECT-type E3 ubiquitin ligase in yeast and is involved in several important cellular processes including transcription regulation, cell cycle progression, mRNA export, and rRNA maturation (9 - 11). More importantly, dysfunction of the Huwe1 (a homolog of yeast Tom1) has been linked with multiple types of cancer and intellectual impairment, and Huwe1 has become a novel therapeutic target for the treatment of cancer (12, 13).

Ubiquitination is a reversible and dynamic PTM that can be counteracted by another set of enzymes, namely deubiquitinating enzymes (Dubs). Dubs play important roles in the ubiquitin pathway by processing Ub precursors, editing or rescuing Ub conjugates, reversing the ubiquitination of substrate proteins, and regenerating monoubiquitin from unanchored ubiquitin oligomers (14, 15). As a deubiquitinating enzyme, Ubp14 in *Saccharomyces cerevisiae* is the major contributor to unanchored (not conjugated to substrate protein) polyubiquitin disassembly and protein deubiquitination (16). Studies with *Arabidopsis* revealed that Ubp14 is conserved in plants and has a similar role in ubiquitin recycling and is required for embryo development, endoreduplication, and auxin response (17 - 19). The deletion of Usp5 (also called isopeptidase T, IsoT), a yeast Ubp14 ortholog in human, also results in the accumulation of polyUb chains (20, 21) and has been implicated in several diseases, especially cancer, by stabilizing different target proteins, such as c-Maf protein, FoxM1, and p53 protein (21, 22).

In addition to ubiquitination/deubiquitination, acetylation is also an important PTM. Acetylation of histone and non-histone proteins involves the transfer of an acetyl group from acetyl coenzyme A to a lysine amino acid in target substrates (23), and this transfer is catalyzed by a set of enzymes called acetyltransferase (24). Since yeast Gcn5 was identified as an acetyltransferase by Brownell and colleagues in 1996 (25), Gcn5 and its homologs have been the most extensively studied acetyltransferases (26). The Spt-Ada-Gcn5-acetyltransferase (SAGA) complex, which includes 19 core subunits, is the largest histone acetyltransferase complex, and Gcn5 serves as the acetyltransferase subunit and is critical for chromatin modification and epigenetic landscape (27, 28). Moreover, Gcn5 regulates multiple biological events in different organisms. In *Arabidopsis*, Gcn5 has been implicated in embryogenesis, root growth, leaf and flower development (29), and the dysfunction of Gcn5 has been associated with many animal diseases such as neurological diseases, cancer, and metabolic and autoimmune disorders (30). The functions of Gcn5 have also been intensively studied in fungi, and increasing evidence demonstrates vital roles for Gcn5 in stress response, fungal development, and pathogenesis (31). For example, our previous studies showed that an imbalance in the Gcn5 activity treated with phenazine-1-carboxamide secreted from *Pseudomonas piscium* or Gcn5 depletion led to defects in *Fusarium graminearum* infection of the host plant (32, 33).

Fusarium head blight (FHB), a devastating disease of small-grain cereal crops worldwide, is primarily caused by the filamentous ascomycete fungus *F. graminearum* (34, 35). In addition to the economic costs of FHB, the mycotoxins including zearalenone and deoxynivalenol (DON) produced by *F. graminearum* are harmful to animals and humans (36, 37). However, efficient management strategies for preventing FHB are not available yet, due to limited resistant cultivars. Our previous study showed that host-induced gene silencing of important genes associated with *F. graminearum* virulence increased disease resistance in wheat (38). Therefore, a better understanding of the molecular mechanisms associated with *F. graminearum* virulence should provide new potential targets and facilitate antifungal development. The role of acetyltransferases, particularly Gcn5, in numerous cellular processes has been extensively studied in recent decades, but little is known about how these enzymes are regulated. In this study, our findings revealed that Tom1 functioned as a novel E3 ligase of Gcn5. Tom1 interacted with Gcn5 and accelerated its degradation via promoting its ubiquitination. Moreover, Ubp14 was first identified as the deubiquitinating enzyme of Gcn5 and was found to stabilize Gcn5 by reducing the Gcn5 ubiquitination level. Thus, the antagonistic functions of Tom1 and Ubp14 in Gcn5 homeostasis appear to play critical roles in the control of Gcn5-mediated virulence in *F. graminearum*.

## RESULTS

### E3 ubiquitin ligase Tom1 interacts with Gcn5

**Fig 1:**
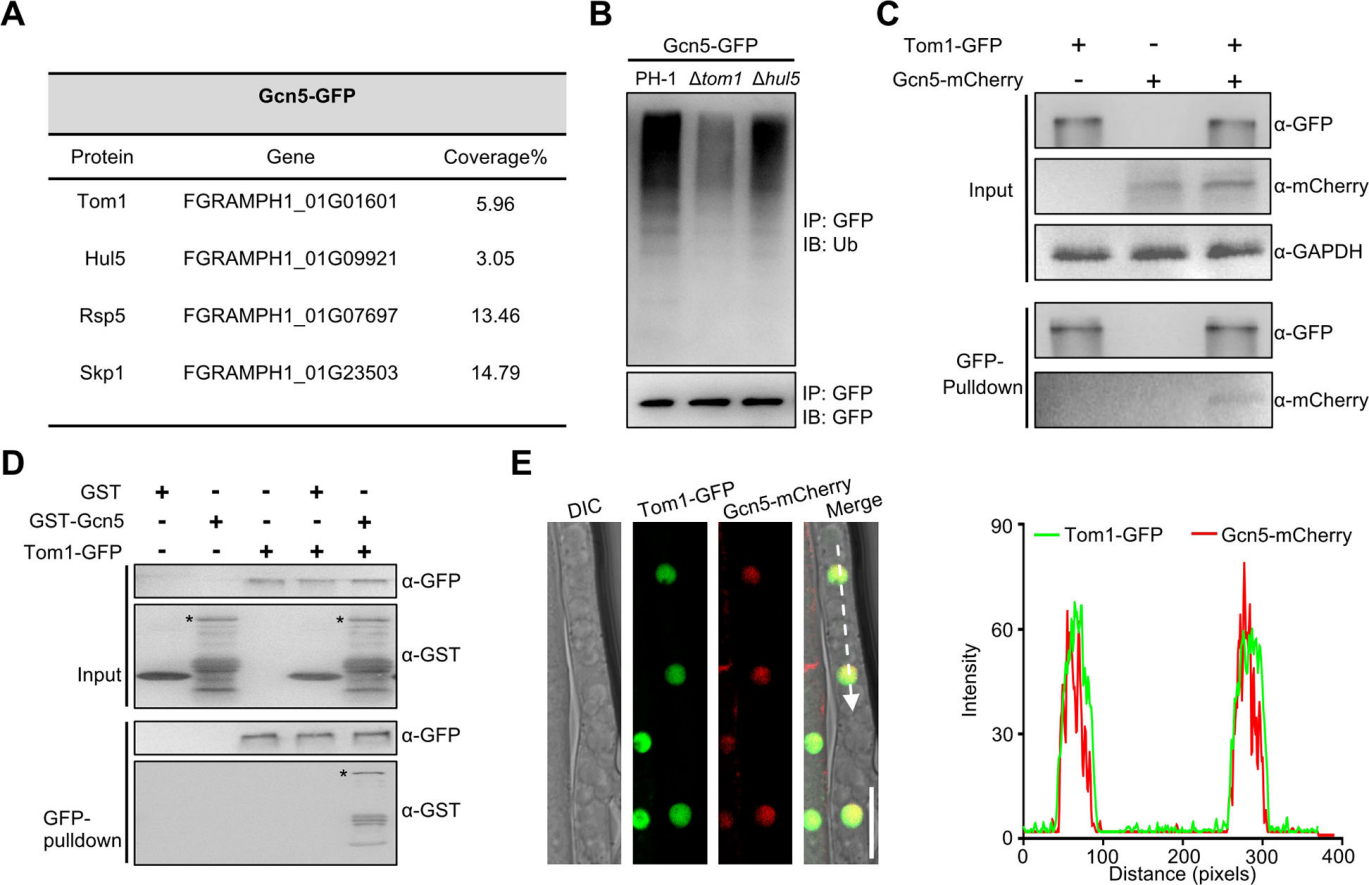
The E3 ubiquitin ligase Tom1 interacts with Gcn5 and regulates its ubiquitination level. (**A**) Putative Gcn5-interacting E3 ubiquitin ligases identiﬁed by afﬁnity puriﬁcation. (**B**) Western blot analysis of Gcn5 ubiquitination in strains expressing Gcn5-GFP on either a wild-type, Δ*tom1*, or Δ*hul5* background. Gcn5-GFP was immunoprecipitated with anti-GFP agarose beads from total proteins. Ubiquitinated and basal Gcn5 proteins were detected by the anti-ubiquitin and anti-GFP antibodies, respectively. (**C**) Western blot showing the interaction between Tom1 and Gcn5 in a co-immunoprecipitation assay. Total proteins isolated from the strains bearing Tom1-GFP and/or Gcn5-mCherry (input), and the proteins eluted from the anti-GFP beads (elution) were detected using anti-GFP and anti-mCherry antibodies, respectively. GAPDH was detected as the internal control. (**D**) Pull-down analysis of the interaction between Tom1 and Gcn5. Tom1-GFP immunoprecipitated from the lysate of the strain expressing Tom1-GFP using anti-GFP agarose beads was incubated with the glutathione *S*-transferase (GST)-tagged Gcn5 purified from *E. coli*. The precipitated complex was analyzed by immunoblotting using an anti-GFP or anti-GST antibody. Asterisks indicate the full-length GST-Gcn5 protein. (**E**) Hyphae were harvested from an *F. graminearum* transformant expressing Tom1-GFP and Gcn5-mCherry. Micrograph (left panel) and line scan graph (right panel) are generated at the indicated position (arrow) showing the co-localization between Tom1-GFP (green) and Gcn5-mCherry (red) in the hyphae of *F. graminearum*. Bar = 10 µm. DIC, differential interference contrast.

We had previously found that Gcn5 was ubiquitinated and subsequently degraded by the proteasome in *F. graminearum* (33). These findings implied that Gcn5 was a substrate of an unknown ubiquitin E3 ligase(s) and deubiquitinating enzyme(s). To identify the Gcn5-related E3 and Dub, we performed an affinity capture assay with Gcn5-green fluorescent protein (GFP) as the bait. The mass spectrometric data revealed that the core subunits of the SAGA complex, such as Ada3 (*FGRAMPH1_01G01875*), Spt8 (*FGRAMPH1_01G11345*), and Sgf73 (*FGRAMPH1_01G17803*) were identified as the putative Gcn5-interacting proteins, demonstrating the reliability of our affinity capture assay (Table S1). Interestingly, four E3 ubiquitin ligases, Tom1, Hul5, Rsp5, and Skp1, were identified as putative Gcn5-interacting proteins (Fig. 1A; Table S1). To identify the true E3 ligase of Gcn5, the ubiquitination level of Gcn5 was examined after the deletion of different E3 ubiquitin ligases. To this end, gene constructs to replace each E3 ubiquitin ligase were generated using a double-joint PCR approach (39) and transformed into the wild-type strain of *Fusarium gramineraum,* PH-1 expressing Gcn5-GFP. Δ*tom1*::Gcn5-GFP and Δ*hul5*::Gcn5-GFP strains were successfully obtained (Fig. S1), but we failed to identify positive gene-knockout mutants of two other E3 ubiquitin ligases, even after screening more than 90 ectopic transformants in three different transformation experiments. Considering the high gene deletion efficiency in *F. graminearum*, failing to isolate their mutants suggests that the E3 ligase Rsp5 and Skp1 might be essential genes (40, 41). Total proteins of PH-1::Gcn5-GFP, Δ*tom1*::Gcn5-GFP, and Δ*hul5*::Gcn5-GFP were isolated, followed by immunoprecipitation (IP) assay using anti-GFP agarose beads, and an immunoblot analysis with an anti-Ub antibody was performed. As shown in Fig. 1B, ubiquitination signals of the Gcn5 protein were significantly reduced in Δ*tom1*::Gcn5-GFP cells compared with PH-1::Gcn5-GFP cells, while the deletion of Hul5 did not significantly affect the ubiquitination level of the Gcn5 protein, suggesting that Tom1 but not Hul5 could participate in the ubiquitination process of Gcn5 in *F. graminearum*.

As Tom1 could act as a ubiquitin E3 ligase for Gcn5, we hypothesized that Tom1 interacts with Gcn5. To test this hypothesis, a strain bearing Tom1-GFP and Gcn5-mCherry was generated, and a Co-IP assay was performed. As expected, Gcn5-mCherry was detected in proteins co-purified with Tom1-GFP but not in negative controls (Fig. 1C), verifying the interaction between Tom1 and Gcn5. Pull-down assay results further confirmed this interaction (Fig. 1D). Previous study showed that Gcn5 was localized in the nucleus (33), and we found that Tom1 co-localized with Gcn5 in the nucleus (Fig. 1E). These findings suggest that Tom1 interacts with Gcn5 in the nucleus and subsequently ubiquitinates Gcn5 in *F. graminearum*.

### Tom1 targets Gcn5 for degradation

**Fig 2:**
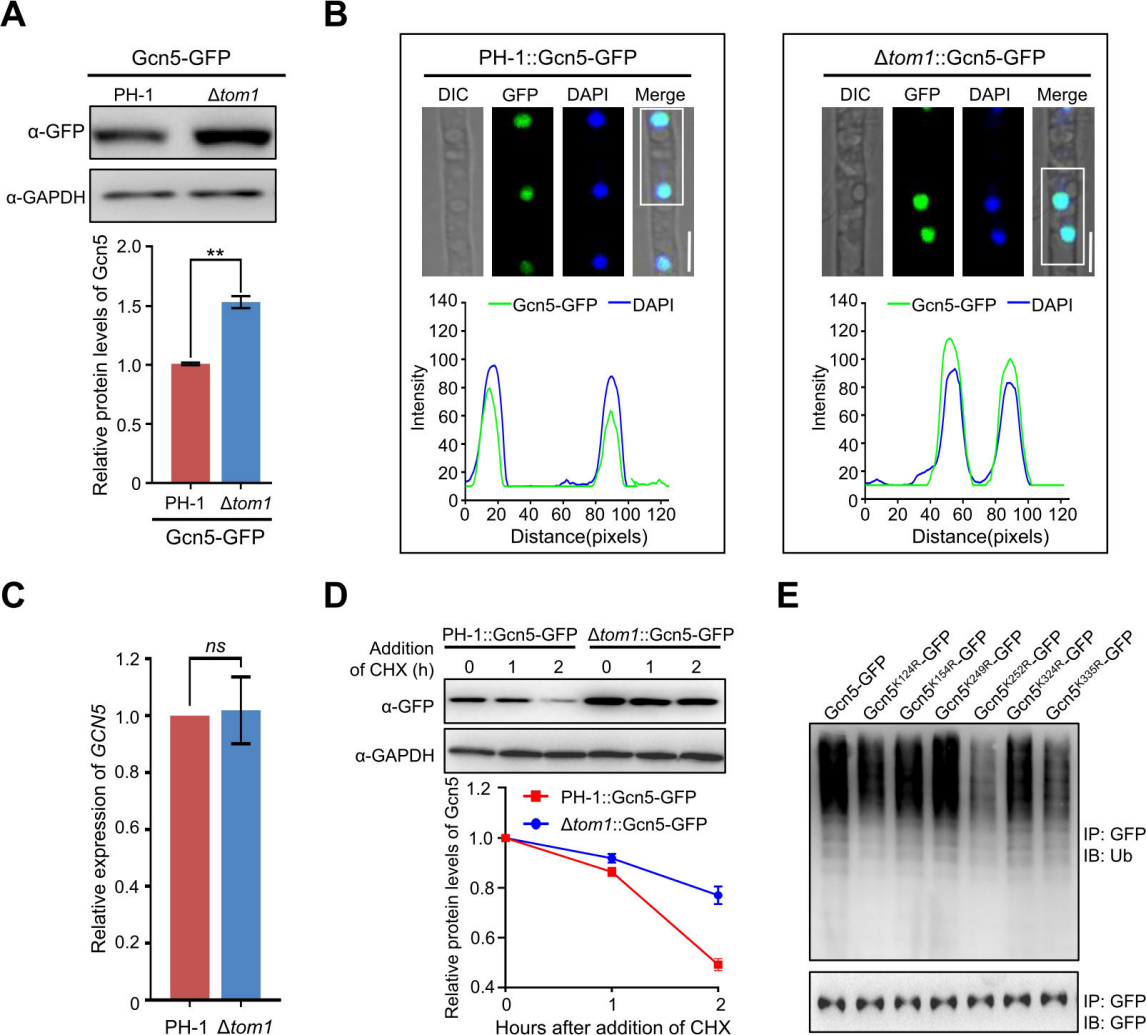
Regulation of Gcn5 protein stability mediated by the E3 ubiquitin ligase Tom1. (**A**) Western blot analysis of Gcn5 protein level in wild-type and Δ*tom1* mutant cells expressing Gcn5-GFP with an anti-GFP antibody. GAPDH was detected as an internal control (upper panel). The band intensities were quantified with ImageJ software (lower panel). (**B**) Fluorescence signals of Gcn5-GFP were measured in the wild-type strain and the Δ*tom1* mutant (upper panel), and the intensity of Gcn5-GFP was quantified with ImageJ (lower panel). Bar = 5 µm. (**C**) Bar charts showing the relative abundance of *GCN5* transcripts in the wild type or the Δ*tom1* mutant using qRT-PCR. (**D**) Western blot analysis showing Gcn5 degradation profiles in the indicated strains after the addition of cycloheximide (CHX) (upper panel). Densitometric quantification of Gcn5 relative to the amount at time 0 is shown in the lower panel. (**E**) Western blot showing the ubiquitination profiles of Gcn5 in the wild type and the strains with a single mutation in the ubiquitination sites. Error bars indicate standard deviation (SD) from three independent experiments. Statistical analysis was performed using Student’s *t*-test. The double asterisks indicate statistical significance with *P* < 0.01. *ns* = no significant difference.

Ubiquitination is an important factor in determining protein degradation, and our results demonstrated that Tom1 interacted with Gcn5 and modulated its ubiquitination process. Therefore, we wondered whether the loss of Tom1 exerts an effect on the protein level and stability of Gcn5. To test this hypothesis, the protein level of Gcn5 in PH-1 or Δ*tom1* mutant was further analyzed by immunoblot analysis. As shown in Fig. 2A, the protein level of Gcn5 increased approximately 1.5-fold in the Δ*tom1* mutant compared with that in the PH-1 strain. Consistent with this observation, the relative fluorescence intensity of Gcn5-GFP was also increased after the deletion of Tom1 (Fig. 2B). To further explore the underlying mechanisms of Tom1 in regulating the protein level of Gcn5, the mRNA level of *GCN5* was measured in the PH-1 strain and Δ*tom1* mutant via quantitative real-time PCR (qRT-PCR), and we found that the *GCN5* mRNA level in the Δ*tom1* mutant was comparable to that in the PH-1 strain (Fig. 2C), ruling out the possibility that the elevated protein level of Gcn5 in the Δ*tom1* mutant was caused by the up-regulation of *GCN5* transcription. Since ubiquitination has been closely associated with protein degradation, the degradation rate of Gcn5 after Tom1 inactivation was investigated. To this end, the degradation profiles of Gcn5 in the PH-1 strain and Δ*tom1* mutant were detected after treatment with the protein synthesis inhibitor cycloheximide (CHX). Notably, we found that when Tom1 was knocked out, the degradation of Gcn5 was delayed (Fig. 2D). Collectively, these findings suggest that Tom1 interacts with Gcn5 and targets Gcn5 for ubiquitination and subsequent degradation in *F. graminearum*.

Because Gcn5 can be ubiquitinated, the ubiquitination sites of Gcn5 are determined in this study. To this end, Gcn5-GFP was purified using anti-GFP agarose beads and then subjected to mass spectrometric analysis. Six putative ubiquitination sites, namely, K124, K154, K249, K252, K324, and K335, were identified (Fig. S2). To identify the main ubiquitination site in Gcn5, single-point mutation strains were generated by mutating every single lysine (K) to arginine (R), a residue that cannot be conjugated to Ub. After an immunoprecipitation/immunoblotting (IP/IB) assay, we found that the mutation in K252 led to a reduction in the ubiquitination level of Gcn5, whereas a mutation in other sites showed no significant effect on Gcn5 ubiquitination (Fig. 2E), suggesting that the 252th lysine residue may be the major ubiquitination site in Gcn5.

### Tom1 is critical for plant infection of *F. graminearum*

**Fig 3:**
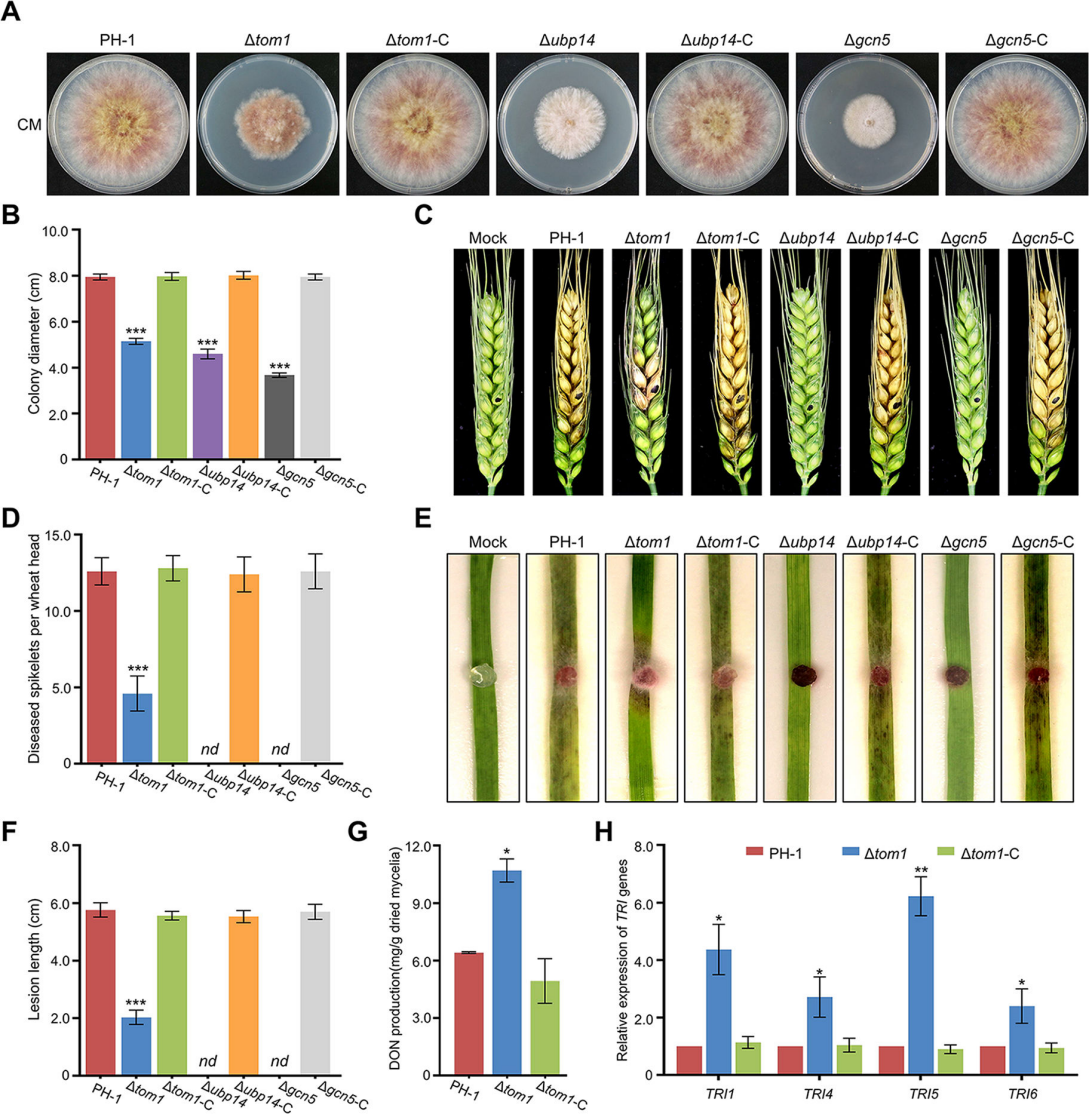
Tom1 and Ubp14 are involved in fungal development and virulence of *F. graminearum*. (**A**) Colony of each strain was grown on complete medium (CM) for 3 days. (**B**) Bar charts showing the colony diameters of the indicated strains. (**C**) Flowering wheat heads were inoculated with mycelial plugs from the indicated strains, and the number of diseased spikelets per wheat head was measured at 14 days post-inoculation (dpi) (**D**). (**E**) Mycelial plugs of the indicated strains were used to inoculate 7-day-old wheat seedlings, and the lesion lengths were examined at 7 dpi (**F**). (**G**) Bar charts showing DON production in 7-day-old TBI cultures of the wild-type PH-1, Δ*tom1*, and corresponding complementation strain (Δ*tom1*-C). (**H**) Bar charts showing the relative transcript abundance of *TRI* genes in the wild type or Δ*tom1* mutant via qRT-PCR. Error bars indicate SD from three independent experiments. Statistical analysis was performed by Student’s *t*-test. *P < 0.05, **P < 0.01, ***P < 0.001. *nd* = not detected.

Previously, we had found that Gcn5 was required for vegetative growth and fungal virulence in *F. graminearum* (32, 33). Similar results were obtained in this study (Fig. 3A through F). Since the E3 ubiquitin ligase Tom1 could mediate the ubiquitination status of Gcn5 and further modulate its degradation and protein level, we wondered whether the loss of Tom1 also affects the fungal development and virulence of *F. graminearum.* To this end, the Δ*tom1* mutant was generated using *F. graminearum* wild-type strain PH-1 as the host strain. The Δ*tom1* mutant and corresponding complementation strain Δ*tom1*-C, together with the wild-type strain PH-1, were inoculated onto complete medium (CM), and we found that the loss of Tom1 led to a marked reduction in hyphal growth, whereas the growth of Δ*tom1*-C was not different from that of PH-1 after inoculation at 25°C for 3 days (Fig. 3A and B). Additionally, the pathogenicity of *F. graminearum* on flowering wheat heads and wheat leaves was reduced after the loss of Tom1 (Fig. 3C through F). The defects in virulence were recovered in Δ*tom1*-C, reaching levels similar to those in the wild type (Fig. 3C through F). Moreover, we found that in contrast to the reduction in DON production detected in Δ*gcn5* (32, 33), Δ*tom1* produced higher levels of DON than the wild-type strain PH-1 after incubation in trichothecene biosynthesis induction (TBI) medium for 1 week (Fig. 3G). As the key virulence factor, the mycotoxin DON produced by *F. graminearum* is synthesized in a specific cellular region called the toxisome, which contains trichothecene synthases encoded by the *TRI* genes (42). To confirm the impact of Tom1 on toxin production, the transcript abundance of four trichothecene biosynthesis-related genes, *TRI1*, *TRI4*, *TRI5*, and *TRI6*, were measured in the PH-1 strain and Δ*tom1* mutant via qRT-PCR. Consistent with the increased DON production and protein level of Gcn5 in the Δ*tom1* mutant, the transcriptional abundances of these four genes were significantly increased in Δ*tom1* compared to those in PH-1 (Fig. 3H). These results suggested that not only Gcn5 deletion mutants but also transformants with enhanced Gcn5 protein level caused by the loss of E3 ubiquitin ligase Tom1 resulted in attenuated virulence of *F. graminearum.* Moreover, the Gcn5^K252R^ point mutation strain reduced the virulence and increased the DON production, which were similar to those in Δ*tom1* mutant (Fig. S3). Together, these results indicate that Tom1 is important for DON production and fungal virulence by mediating the proper ubiquitination of Gcn5.

### Ubp14 interacts with Gcn5

**Fig 4:**
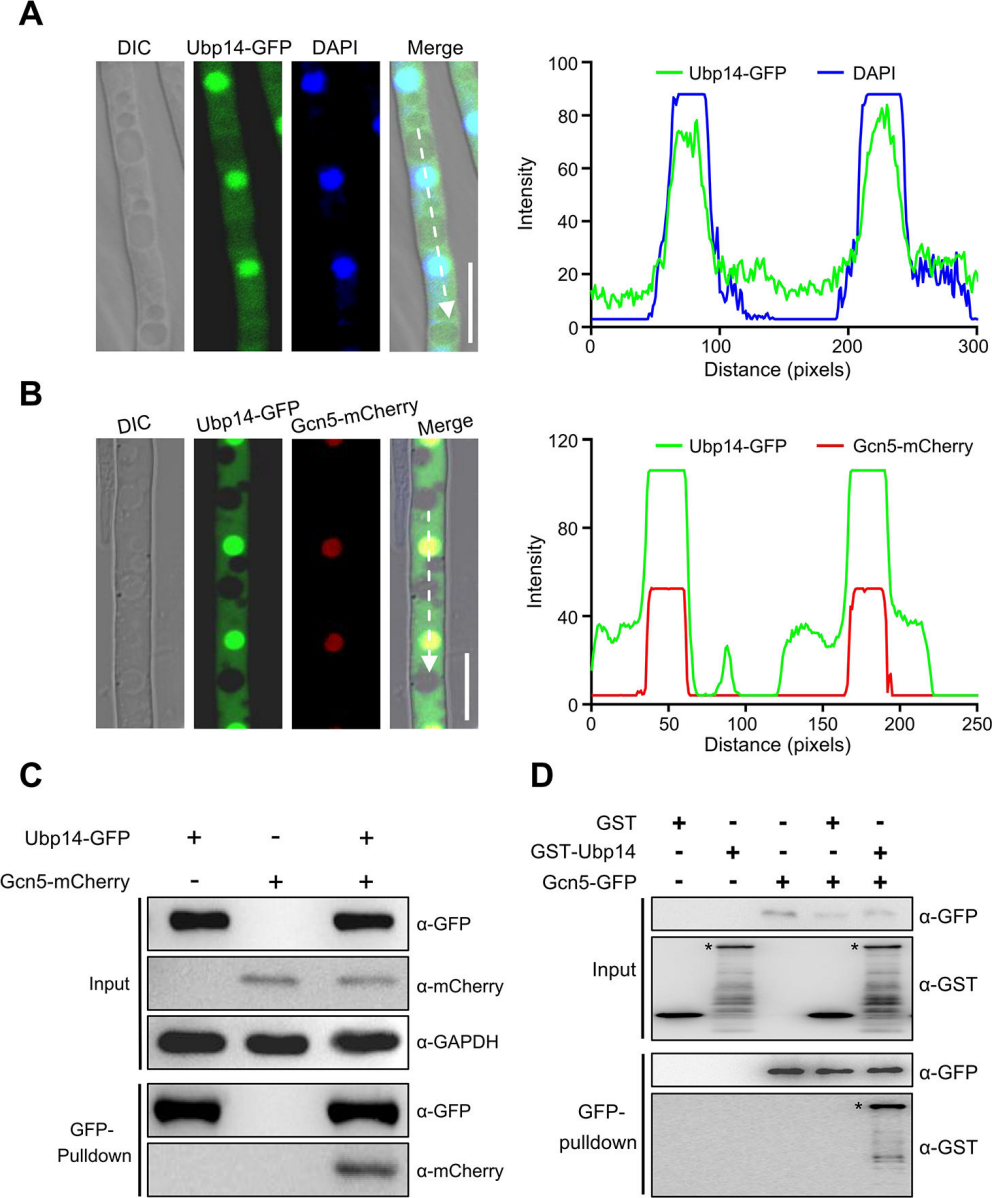
Ubp14 interacts with Gcn5 in *F. graminearum.* (**A**) Micrographs and their corresponding line scan graphs showing the localization of Ubp14 in hyphae of *F. graminearum*. 4′,6-diamidino-2-phenylindole (DAPI) was used to visualize the nuclei. A line scan graph was generated at the indicated position (arrow) to show the relative localization of Ubp14-GFP (green) and DAPI (blue). Bar = 10 µm. (**B**) Hyphae were harvested from an *F. graminearum* transformant expressing Ubp14-GFP and Gcn5-mCherry. Micrograph and line scan graphs generated at the indicated position (arrow) showing the partial co-localization between Ubp14-GFP (green) and Gcn5-mCherry (red) in the hyphae of *F. graminearum*. Bar = 10 µm. (**C**) Western blot showing the interaction between Ubp14 and Gcn5 after a co-immunoprecipitation (Co-IP) assay. Total proteins isolated from the strains bearing Ubp14-GFP and/or Gcn5-mCherry (input), and the proteins eluted from the anti-GFP beads (elution) were detected using anti-GFP and anti-mCherry antibodies, respectively. GAPDH was detected as the internal control. (**D**) Pull-down analysis of the interaction between Ubp14 and Gcn5. Gcn5-GFP immunoprecipitated from the lysate of the strain expressing Gcn5-GFP with anti-GFP antibody beads was incubated with GST-tagged Ubp14 purified from *E. coli*. The precipitated complex was analyzed by immunoblotting using an anti-GFP or anti-GST antibody. Asterisks indicate the full-length GST-Ubp14 protein.

In addition to E3 ubiquitin ligases, a deubiquitinating enzyme (*FGRAMPH1_01G06337*, designated *UBP14*) was also identified to interact with Gcn5 in two independent biological replicates (Table S1). To verify the interaction between Ubp14 and Gcn5 in *F. graminearum*, first, a strain expressing Ubp14-GFP was generated and the localization of Ubp14 was detected via confocal microscopy. As shown in Fig. 4A, Ubp14-GFP was partially co-localized with DAPI, a dye used to stain the nuclei. Additionally, certain fluorescence signals were also observed in the cytoplasm. Since Gcn5 was localized in the nucleus (33), suggesting that Gcn5 may co-localized with Ubp14 in the nucleus. To test this hypothesis, a strain bearing Ubp14-GFP and Gcn5-mCherry was generated. Our results demonstrated that Gcn5-mCherry signals were clearly co-localized with Ubp14-GFP in the nucleus (Fig. 4B). Co-IP assays and pull-down assays were further conducted to confirm the interaction between Ubp14 and Gcn5 (Fig. 4C and D). To further confirm these results, cytoplasmic and nuclear fractions were isolated from the strains bearing Ubp14-GFP and Gcn5-mCherry, respectively, and subsequent Co-IP assays were performed. Results showed that Ubp14-Gcn5 interaction was only detected in the nuclear fraction (Fig. S4). Collectively, these results suggest that Ubp14 interacts with Gcn5 in the nucleus in *F. graminearum.*

### Ubp14 exhibits deubiquitination activity

**Fig 5:**
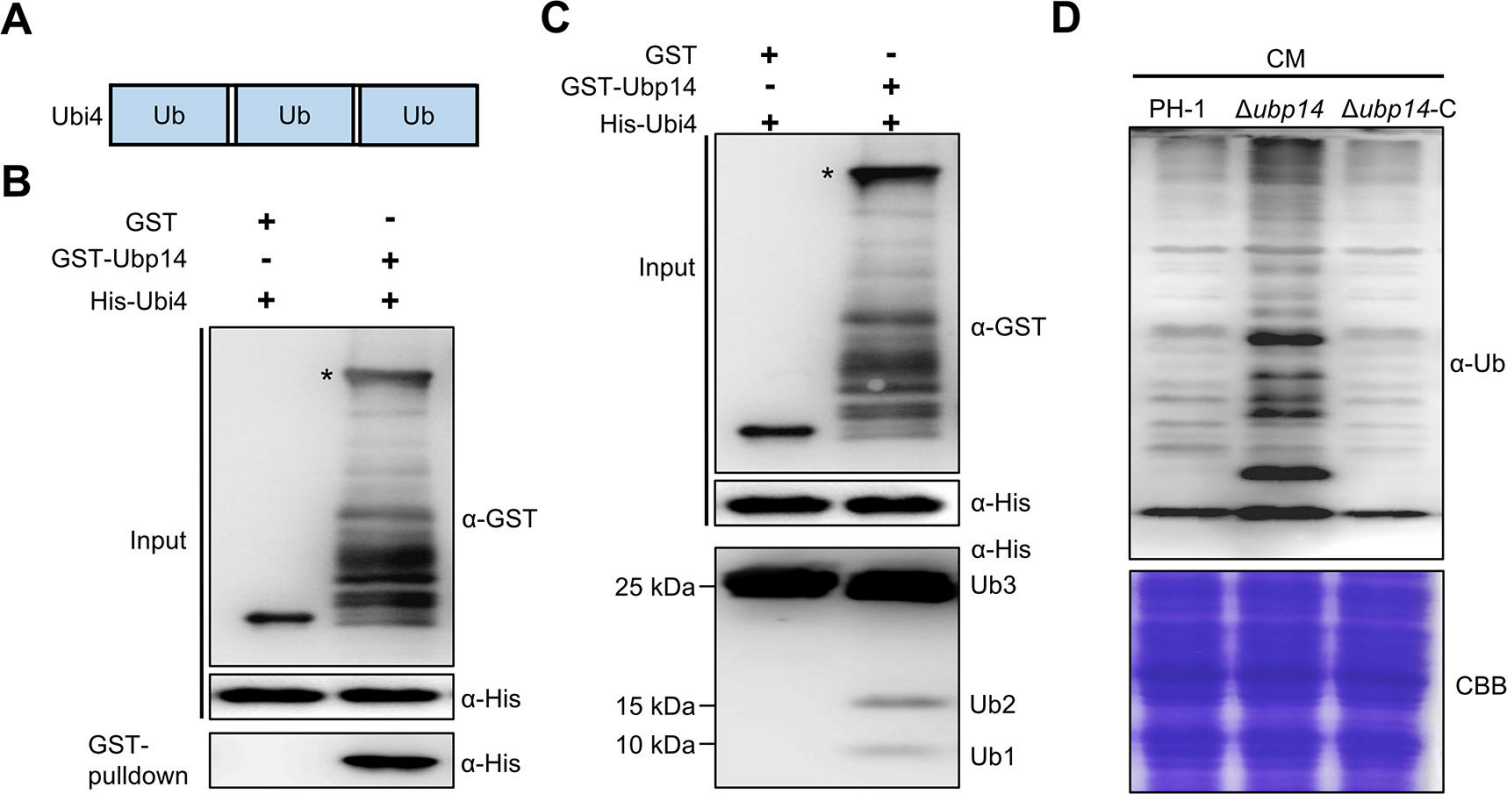
Ubp14 exhibits deubiquitination activity in *F. graminearum*. (**A**) Domain structure of the Ubi4 in a GST pull-down assay. (**B**) GST or GST-Ubp14 immobilized on GST beads was incubated with His-Ubi4 proteins. The beads were washed and pelleted for immunoblotting with an anti-His antibody. Asterisks indicate the full-length GST-Ubp14 protein. (**C**) Ubp14 showed deubiquitination activity *in vitro*. GST-Ubp14, but not the GST protein alone, cleaved Ubi4 containing three Ubs into a di-Ub (Ub2) and a mono-Ub (Ub1) product. Asterisks indicate the full-length GST-Ubp14 protein. (**D**) Ubp14 showed deubiquitination activity *in vivo*. After growth in CM for 12 hours, total proteins in the tested strains were subjected to SDS-PAGE gel and immunoblotted with an anti-ubiquitin antibody to measure the ubiquitination levels. Coomassie brilliant blue (CBB) staining was used during protein loading.

In yeast, the Ubp14 protein exhibits deubiquitination activity (15, 16). Ubp14 in *F. graminearum* shares 33.62% amino acid identity with *S. cerevisiae* Ubp14 and carries a Ubp (ubiquitin-specific processing protease) domain, indicating that *F. graminearum* Ubp14 may also exhibit potential deubiquitination activity similar to that of *S. cerevisiae* Ubp14. To test this hypothesis, an enzymatic activity assay was performed using the trimeric polyubiquitin protein Ubi4 (*FGRAMPH1_01G10777*), which harbors three Ub_domain repeats (1–72 aa, 77–148 aa, and 153–224 aa) (Fig. 5A). His-Ubi4, glutathione *S*-transferase (GST)-Ubp14, and GST alone were separately expressed in *Escherichia coli*. After purification, our results revealed that GST-Ubp14 but not GST alone was able to bind to His-Ubi4 in the GST pull-down assay (Fig. 5B), suggesting that Ubp14 interacted with Ubi4 *in vitro*. Additionally, immunoblot analysis revealed that GST-Ubp14 but not GST protein alone was able to cleave the *UBI4*-encoded tri-ubiquitin chain into di- and mono-Ub (Fig. 5C), suggesting that Ubp14 possessed deubiquitination activity *in vitro*. To test the deubiquitination activity of Ubp14 *in vivo*, the total proteins of Δ*ubp14* and the wild-type strain PH-1 were isolated, and ubiquitinated proteins were detected with an anti-Ub antibody. According to Fig. 5D, the wild-type strain had lower levels of total ubiquitinated proteins than the strain with the depletion of Ubp14. To further confirm that the defect in the abnormal ubiquitination status of total proteins in the Δ*ubp14* mutant was directly caused by the loss of the *UBP14* gene, the complementation strain Δ*ubp14*-C was generated. We found that Δ*ubp14*-C restored the defects and showed a ubiquitination pattern similar to that of PH-1. These results suggest that Ubp14 has deubiquitination activity *in vitro* and *in vivo*.

### Ubp14 maintains Gcn5 protein stability

**Fig 6:**
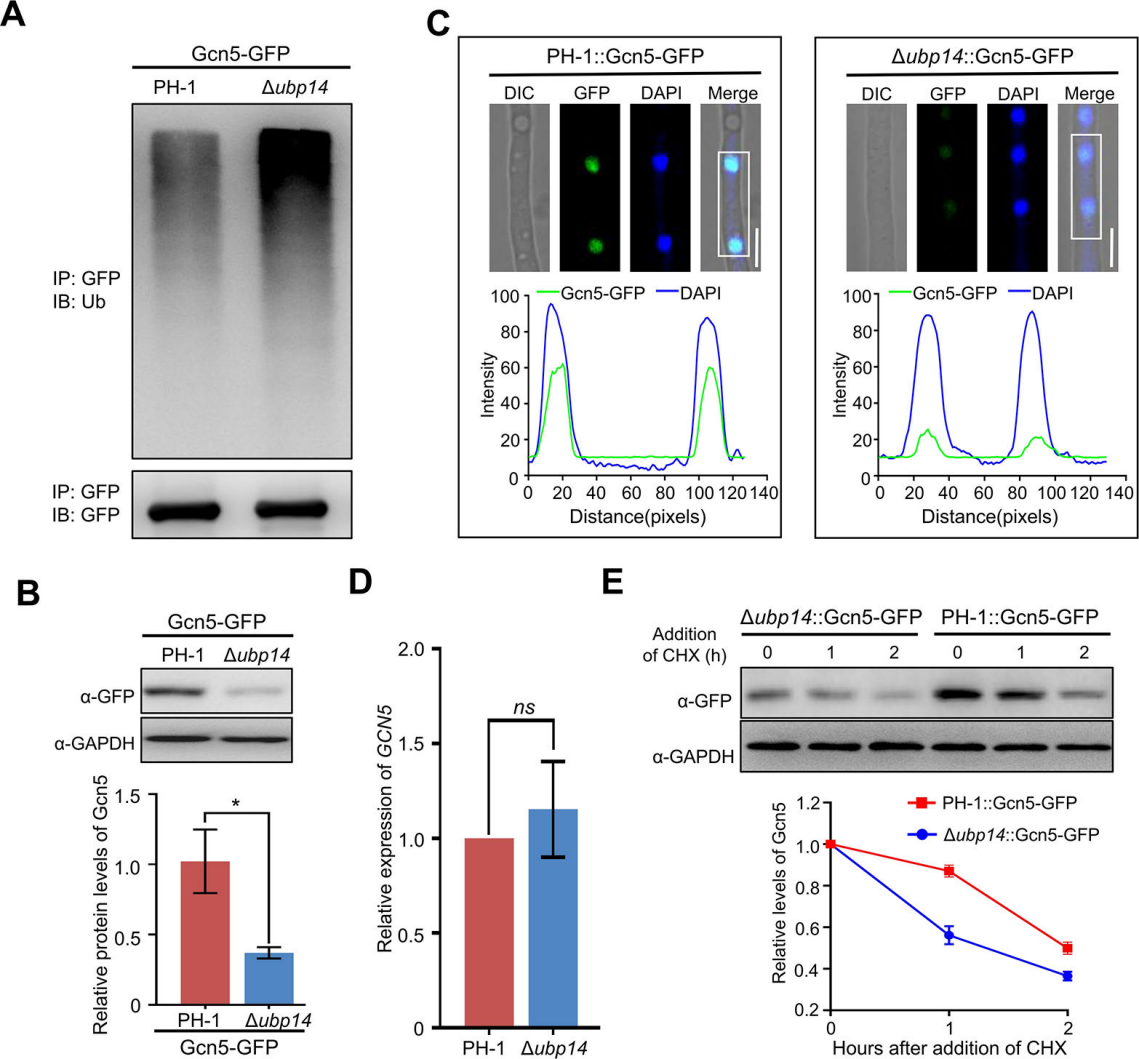
Ubp14 regulates the protein stability of Gcn5 in *F. graminearum.* (**A**) Western blot analysis of Gcn5 ubiquitination in strains expressing Gcn5-GFP on either a wild-type or Δ*ubp14* background. Gcn5-GFP was isolated and then immunoprecipitated with anti-GFP agarose beads from solubilized proteins. Ubiquitinated and basal Gcn5 proteins were detected by anti-ubiquitin and anti-GFP antibodies, respectively. (**B**) Western blot analysis of Gcn5 protein level in the wild type and Δ*ubp14* mutant expressing Gcn5-GFP with an anti-GFP antibody, and GAPDH was detected as the internal control (upper panel). The band intensities were quantified with ImageJ software (lower panel). (**C**) Fluorescence signals of Gcn5-GFP were detected in the wild-type strain and the Δ*ubp14* mutant (upper panel), and the intensity of Gcn5-GFP was quantified with ImageJ software (lower panel). Bar = 5 µm. (**D**) Bar charts showing the relative abundance of *GCN5* transcripts in the wild type or Δ*ubp14* mutant via qRT-PCR. (**E**) Western blot analysis showing Gcn5 degradation profiles in the indicated strains after the addition of cycloheximide (CHX) (upper panel). Quantification of Gcn5 protein level relative to the amount at time 0 is shown in the lower panel. Error bars indicate SD from three independent experiments. Statistical analysis was performed via Student’s *t*-test. A single asterisk indicates statistical significance at *P* < 0.05. *ns* = no significant difference.

Ubp14 showed deubiquitination activity and interacted with Gcn5, suggesting that Ubp14 may deubiquitinate Gcn5 in *F. graminearum.* First, the ubiquitination pattern in the wild-type and Δ*ubp14* mutant strains was tested. To this end, PH-1 and the Δ*ubp14* mutant expressing Gcn5-GFP were generated and then total proteins of these two strains were isolated, and Gcn5-GFP proteins were immuoeprecipitated (IP) using anti-GFP agarose beads. A subsequent immunoblot analysis with an anti-Ub antibody showed that the deletion of Ubp14 markedly increased the ubiquitination level of Gcn5 (Fig. 6A), suggesting that Ubp14 is required for the removal of ubiquitin from Gcn5. Moreover, according to Fig. 6B, the protein level of Gcn5 was significantly reduced after the depletion of Ubp14. Consistently, the fluorescence intensity of Gcn5-GFP was much weaker after the loss of Ubp14 (Fig. 6C).

According to Fig. 6D, the *GCN5* mRNA level in the Ubp14-depleted strain remained largely unchanged compared to that in the PH-1 strain, indicating that the effect of Ubp14 on the Gcn5 protein level was not mediated through transcriptional regulation. Since the loss of Ubp14 induced hyperubiquitination of Gcn5, we next wondered whether Ubp14 regulates the protein stability of Gcn5. Our results showed that Gcn5 degraded more rapidly in the Δ*ubp14* mutant than in the wild-type background (Fig. 6E), suggesting that contrary to Tom1, the loss of Ubp14 promoted the protein degradation of Gcn5. Taking these results together, we concluded that Ubp14 targets Gcn5 and inhibits its ubiquitination, thus stabilizing Gcn5 in *F. graminearum.*

### Ubp14 is critical for plant infection by regulating DON production, autophagy, and penetration

Since Gcn5 is required for vegetative growth and plant infection (32, 33), we wondered whether Ubp14 also regulated the fungal development and virulence of *F. graminearum*. As shown in Fig. 3A and B, the deletion of Ubp14 also led to a reduction in vegetative growth. Additionally, we found that the depletion of Ubp14 led to the loss of *F. graminearum* virulence. No visible disease was caused by the Δ*ubp14* mutant on inoculated wheat spikelets or leaves, but typical scab symptoms were detected in spikelets or leaves inoculated with PH-1 or Δ*ubp14*-C under identical conditions (Fig. 3C through F), demonstrating that *F. graminearum* Ubp14 is important for plant infection.

**Fig 7:**
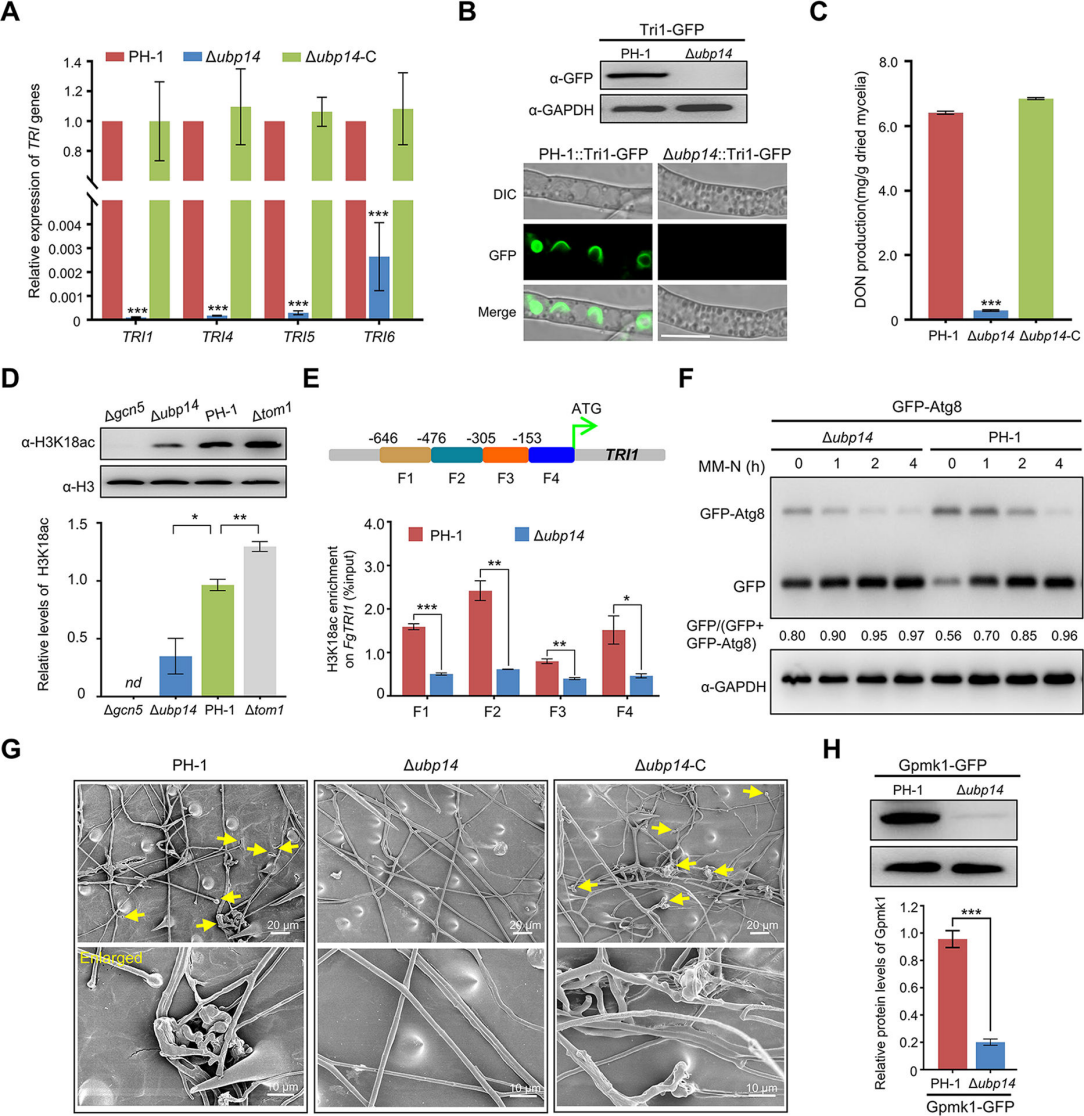
Ubp14 is critical for DON production, autophagy, and penetration in *F. graminearum.* (**A**) Bar charts showing the relative transcriptional abundance of *TRI* genes in the wild type or Δ*ubp14* mutant using qRT-PCR. (**B**) Western blot analysis of Tri1 protein level in the wild type and Δ*ubp14* mutant expressing Tri1-GFP with an anti-GFP antibody (upper panel). Fluorescence signals of Tri1-GFP were measured in the wild-type strain and the Δ*ubp14* mutant after incubation for 48 hours in the TBI medium (lower panel). Bar = 10 µm. (**C**) Bar charts showing the levels of DON production in 7-day-old TBI cultures of the wild-type PH-1, Δ*ubp14*, and corresponding complementation strain (Δ*ubp14*-C). (**D**) The effects of indicated mutants on histone acetylation. Anti-H3K18ac and anti-H3 antibodies were used to detect histone acetylation and served as a loading control, respectively. (**E**) Chromatin immunoprecipitation–quantitative PCR (ChIP-qPCR) assays showing the enrichment of H3K18ac at the promoters of the *TRI1* in the indicated strains. (**F**) Western blot showing GFP-Atg8 proteolysis in the wild-type or Δ*ubp14* background using an anti-GFP antibody. The protein expression levels of GAPDH in the tested samples were used as loading controls. (**G**) Wheat glumes were inoculated with mycelial plugs of the indicated strains. The infection structures marked with yellow arrows were observed in the wild-type strain and the Δ*ubp14* complementation strain but not in the Δ*ubp14* mutant. (**H**) Upper panel images showing the protein amount of Gpmk1 in the wild-type strain PH-1, compared to that in the Δ*ubp14* mutant. GAPDH was measured as the internal control. The band intensities were quantified with ImageJ software (lower panel). Error bars indicate SD from three independent experiments. Statistical analysis was performed via Student’s *t*-test. *P < 0.05, **P < 0.01, ***P < 0.001. *nd* = not detected.

Since the deletion of Ubp14 leads to a reduced protein level of Gcn5 and the loss of Gcn5 completely abolished DON biosynthesis (32, 33), we hypothesized that the reduction in virulence in the Δ*ubp14* mutant was partially due to reduced DON production. To test this, transcriptional abundances of four *TRI* genes (*TRI1*, *TRI4*, *TRI5*, and *TRI6*) were determined in Δ*ubp14* and PH-1 using qRT-PCR, and we found that transcriptional levels of these four *TRI* genes were significantly decreased in Δ*ubp14* compared to those in PH-1 (Fig. 7A). In addition, toxisome formation for DON biosynthesis in Δ*ubp14* and PH-1 was detected using Tri1-GFP as an indicator (42). An immunoblot analysis revealed that the Tri1-GFP level was negligible in the mutant, as determined using an anti-GFP antibody (Fig. 7B). In accordance with the immunoblot analysis, the Tri1-GFP was almost undetected after the deletion of Ubp14, whereas strong fluorescence signals were found in spherical- or crescent-shaped toxisomes in the wild-type background after 2 days of incubation in TBI medium (Fig. 7B). Consistent with reduced *TRI* expression and abolished toxisome formation in Δ*ubp14*, the level of DON produced by Δ*ubp14* was approximately 12-fold lower than that of PH-1 (Fig. 7C). Taken together, these results indicate that, similar to Gcn5, Ubp14 is required for toxisome formation and mycotoxin production in *F. graminearum.* In previous studies, we found that Gcn5 was critical for histone acetylation and subsequent induction of gene transcription, especially the acetylation of histone 3K18 (H3K18ac) (32, 33). In mammalian cells, H3K18ac is mainly enriched at promoters of target genes for transcriptional activation (43, 44). To explain how Ubp14 modulates DON biosynthesis, we first checked the level of H3K18ac in the Δ*ubp14* mutant. Consistent with our previous studies (32, 33), H3K18ac was almost undetectable in Δ*gcn5* mutant, while the acetylation level of H3K18 was increased in the Δ*tom1* mutant (Fig. 7D). In accordance with a reduction of Gcn5 protein level, the acetylation level at H3K18 was also reduced after the deletion of Ubp14 (Fig. 7D). To detect whether the downregulation of above *TRI* genes (*TRI1*, *TRI4*, *TRI5*, and *TRI6*) in Δ*ubp14* mutant is due to the changes in histone acetylation levels at these specific gene loci, the enrichment of H3K18ac on the promoters of these *TRI* genes was detected in PH-1 and Δ*ubp14* mutant by chromatin immunoprecipitation–quantitative PCR (ChIP-qPCR) analysis. Results indicated that the enrichments of H3K18ac on the promoter regions of selected *TRI* genes were reduced in the Δ*ubp14* mutant, compared with those in the PH-1 (Fig. 7E; Fig. S5). These results suggested that Ubp14 regulates the transcription of DON biosynthetic genes by alteration of Gcn5-mediated histone acetylation, especially H3K18ac.

Another marked defect in the Δ*gcn5* mutant was abnormal autophagy, and the deletion of Gcn5 strongly promoted the autophagy process (32, 33). Since the deletion of Ubp14 led to a reduction in the Gcn5 protein level, we hypothesized that autophagy may be impaired by the deletion of Ubp14. To test this hypothesis, GFP-Atg8, a commonly used autophagy pathway marker (45), was expressed in the Δ*ubp14* and PH-1, and the autophagy process was determined by measuring GFP-Atg8 proteolysis (46). The breakdown of GFP-Atg8 was determined by measuring the ratio of free GFP to intact GFP-Atg8 plus free GFP. As shown in Fig. 7F, a higher ratio of free GFP was detected in the Δ*ubp14* mutant at each tested time point compared to the wild-type strain, suggesting that similar to Gcn5, Ubp14 acts as a negative regulator of the autophagy process and that the loss of Ubp14 promotes autophagy in *F. graminearum*.

Since Gcn5 modulated the infection structure formation and the loss of Ubp14 led to lost virulence on flowering wheat heads and wheat leaves, indicating that the penetration of *F. graminearum* was impaired after the depletion of Ubp14. Therefore, the penetration ability of Δ*ubp14* was also analyzed via scanning electron microscopy. As expected, typical penetration-related structures were formed on wheat spikelets inoculated by PH-1 and *Δubp14-C* but not the Δ*ubp14* mutant (Fig. 7G). The Gpmk1 mitogen-activated protein kinase (MAPK) pathway is critical for the formation of penetration structures, which is important for the infection of phytopathogens (47 - 49). As the downstream and key component of the Gpmk1 MAPK pathway, the Gpmk1 protein level in the PH-1 and Δ*ubp14* mutant was assessed. In agreement with the microscopy results, the protein level of Gpmk1 was reduced in the Δ*ubp14* mutant compared to the wild-type strain (Fig. 7H), indicating that Ubp14 was involved in the penetration processes of *F. graminearum.* Collectively, these findings suggest that Ubp14 might regulate the protein level of Gcn5 by affecting its ubiquitination level and further modulating multiple cellular processes, including DON production, autophagy, and penetration, which are critical for the *F. graminearum* virulence.

## DISCUSSION

The roles of Gcn5 and its homologs in the regulation of multiple cellular processes, including transcription (25), DNA repair (50), telomere maintenance (51), and nucleosome assembly (52), have been widely studied in different organisms. However, the regulatory network of PTMs that affects Gcn5 is still largely unknown. Lines of evidence presented in this study indicate that the E3 ligase Tom1 and the deubiquitinating enzyme Ubp14 play antagonistic roles in the control of Gcn5 activity. Therefore, the results of the present study extend the understanding of the cross-talk between acetylation and ubiquitination.

In mammalian cells, Gcn5 ubiquitination was regulated by the CRL4^Cdt2^ E3 ligase complex (53), which mainly consists of a scaffold protein Cullin4 (Cul4), the adaptor protein DNA damage-binding protein 1 (Ddb1), and a substrate receptor protein Cdc10-dependent transcript 2 (Cdt2) (54, 55). The homolog of the CRL4^Cdt2^ E3 ligase complex was identified in the *F. graminearum* genome database via BLASTP algorithm with the Cul4, Ddb1, and Cdt2 in humans as queries (Fig. S6A). However, the CRL4^Cdt2^ E3 ligase complex is dispensable for the Gcn5 ubiquitination in this fungus since the ubiquitination level of the Gcn5 protein was not affected in each mutant of CRL4^Cdt2^ E3 ligase complex (Fig. S6B). Here, we reported that the Gcn5 ubiquitination level was significantly decreased on deletion of another E3, namely, Tom1. Consistently, Gcn5 was found to be more stable, and the abundance of the Gcn5 protein was increased in the Δ*tom1* mutant, indicating that Tom1 acted as a new and previously unknown E3 ligase of Gcn5. Other studies have shown that two E3 ubiquitin ligases can act together and target one substrate for degradation (56, 57). In this study, we noticed that although the Gcn5 was more stable in the Δ*tom1* mutant than that in the wild-type strain, the Gcn5 protein was still degraded in the Δ*tom1* mutant, suggesting that other E3 ligases are working together with Tom1 in the regulation of the ubiquitination and stability of Gcn5. In addition to Tom1 and Hul5, Rsp5 and Skp1 were identified as Gcn5-interacting E3 ligases. We cannot rule out that these two E3 ligases, or other undiscovered E3 ligases, may be involved in the ubiquitination and degradation of Gcn5 in *F. graminearum* since these two genes seem to be essential in this fungus, and their effect on Gcn5 could not be determined in this study. Therefore, the identification of other E3 ligases plus any currently unknown E1 and E2 of Gcn5 will complete the ubiquitination pathway of Gcn5.

The *F. graminearum* Ubp14 showed deubiquitination activity *in vivo* and *in vitro* in the present study. Orthologs of Ubp14 in *S. cerevisiae*, *Arabidopsis*, and humans are important for the regeneration of mono-Ub and the disassembly of free polyubiquitin chains derived from ubiquitinated proteins (16 - 20 - 21 - 21), suggesting a conserved function of Ubp14 in ubiquitin recycling and homeostatic maintenance of the ubiquitin pool. The findings presented in this study indicate that Gcn5 is a bona fide target of Ubp14, which is based on four lines of evidence. First, Ubp14 interacted with Gcn5. Second, Ubp14 negatively regulated the ubiquitination of Gcn5. Third, the loss of Ubp14 promoted the degradation of Gcn5 and led to a reduction in the protein level of Gcn5. Finally, the Δ*ubp14* mutant showed defects in DON production, autophagy process, and the formation of penetration structures and further led to the abolishment of *F. graminearum* virulence, similar to the effects observed in the Δ*gcn5* mutant. Because Gcn5 was more unstable and the protein level of Gcn5 was significantly decreased in the Δ*ubp14* mutant, defects in the Δ*ubp14* mutant appeared to stem from the inhibition of Gcn5-mediated acetyltransferase activity. To our knowledge, this is the first report of a deubiquitinating enzyme acting on Gcn5 in filamentous fungi.

Compared to those of ubiquitination, the functions of the deubiquitination process are less understood, especially in filamentous fungi. Previous studies have shown that Usp5, the human ortholog of yeast Ubp14, plays critical roles in different cancers by targeting distinct substrates, such as p53, FoxM1, and β-catenin (22). In this study, our biochemical analysis showed that Ubp14 deubiquitinated Gcn5. As shown in Fig. 5D, the loss of Ubp14 significantly elevated the ubiquitination level of total protein. Moreover, the subcellular localization of Ubp14 was also analyzed in this study, and our microscopy observations showed that Ubp14 localized both to the cytoplasm and the nucleus, whereas Gcn5 was exclusively localized to the nucleus. These results suggest that similar to Usp5, Ubp14 might target a variety of substrates and these substrates might be nuclear localized (such as Gcn5 in this study) or non-nuclear localized. Further, the identification of the target proteins of Ubp14 will help us to better understand how deubiquitinating enzymes contribute to different aspects of fungal development and virulence.

Signals such as heat, osmotic stress, ultraviolet, and nutrition have been shown to regulate the protein ubiquitination (58, 59). For example, nitrogen regulated the ubiquitination of the general amino acid permease Gap1 in *S. cerevisiae* (59). Mammalian target of rapamycin (mTOR) inhibition on rapamycin treatment significantly increased the total content of ubiquitinated proteins and stimulated ubiquitin proteasome system–mediated proteolysis (60). Once a signal is received, several different signal transduction pathways are involved in the dynamic regulation of ubiquitination and deubiquitination cycle of target proteins. For instance, the E3 ligase Mdm2 is required for the ubiquitination and degradation of the tumor suppressor p53. The mTOR/Akt signaling promotes the Mdm2 translocation to the nucleus, where it targets p53 for degradation (61 - 63). In contrast, the transcription factor Atf3 competes with p53 for Mdm2-mediated ubiquitination and stabilizes p53 by preventing p53 from Mdm2-mediated ubiquitination (64). We previously reported that the ubiquitination level of Gcn5 was dramatically increased on rapamycin treatment or nitrogen starvation in *F. graminearum* (33), suggesting that rapamycin or nitrogen source might serve as signals to regulate the dynamic of ubiquitination/deubiquitination cycle of Gcn5. Here, we found that Tom1-Gcn5-Ubp14 circuit plays an important role in the fungal development and virulence in *F. graminearum.* It implied that signals from fungal development and plant–pathogen interaction process might also regulate the dynamic of ubiquitination status of Gcn5. Therefore, the identification of signals and signal transduction pathways for regulating ubiquitination and deubiquitination of Gcn5 needs to be further understood and addressed.

**Fig 8:**
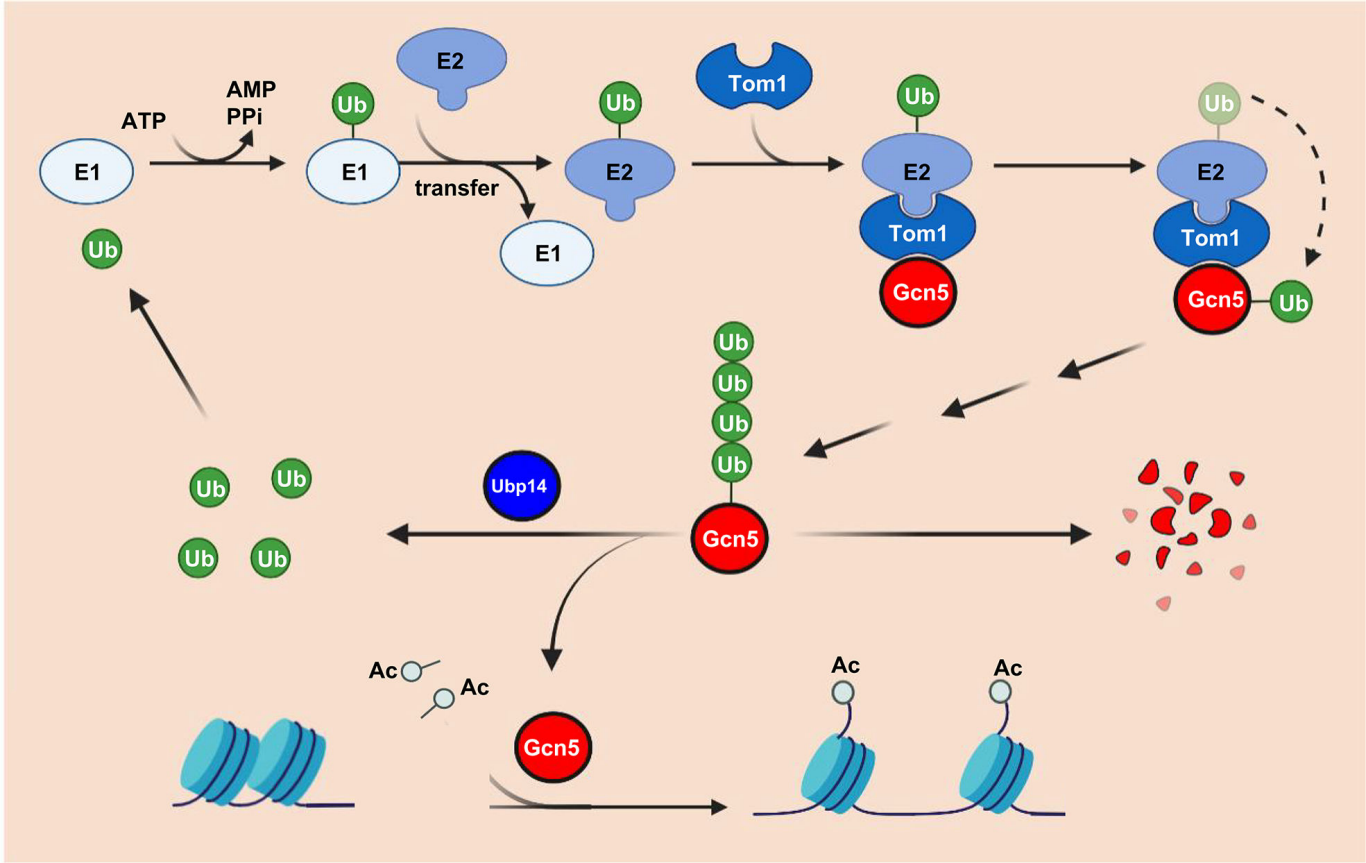
Proposed model for the regulation of fungal virulence by Tom1-Gcn5-Ubp14 signaling in *F. graminearum*. As a histone acetyltransferase, Gcn5 regulates histone acetylation and further modulates *F. graminearum* virulence. The deubiquitinating enzyme Ubp14 interacts with Gcn5 and stabilizes Gcn5 by preventing its ubiquitination. In contrast, the E3 ubiquitin ligase Tom1 promotes the ubiquitination of Gcn5 with the assistance of unknown E1 and E2 and further facilitates the degradation of Gcn5. Ub, ubiquitin; Ac, acetyl group; Ppi, pyrophosphate; E1, Ub-activating enzyme; and E2, Ub-conjugating enzyme.

In addition to E3 ligases and deubiquitinating enzymes, several kinases and methyltransferases have been identified as potential Gcn5-interacting proteins (Table S1). The mechanisms of their actions are yet to be established, but in addition to ubiquitination/deubiquitination, it is likely that the activity of Gcn5 is also modulated by other PTMs in *F. graminearum*. It will be interesting to learn how these different modifications work together to regulate the activity of Gcn5, which will provide a better framework for understanding the true breadth of acetyltransferase functions across a wide range of eukaryotic species. In summary, we propose a possible model showing that Tom1, Gcn5, and Ubp14 coordinately modulate the virulence of *F. graminearum* (Fig. 8), providing novel possibilities and targets to control FHB.

## Materials and Methods

### Strains and growth condition

*UBP14* gene deletion mutant, namely, Δ*ubp14* and other gene deletion mutants were generated in this study using the PH-1, the wild-type strain of *F. graminearum*, as the host strain. The strains used in this study were cultured and stored on potato dextrose agar medium. The liquid trichothecene biosynthesis induction (TBI) medium was used for the induction of DON production (42).

### Generation of mutants, complementation strains, and tag-fusion cassettes

Gene deletion mutants were generated using the strategies described previously (39). Briefly, approximately 1,000-bp upstream and downstream flanking sequences of targeted genes were amplified and were further fused with selection markers, hygromycin phosphotransferase (*HPH*) or nourseothricin (*NTC*) cassettes using the double-joint PCR (Fig. S1). The PCR products were transformed into protoplasts of the host strain following protocols described previously (65, 66). PCR assay was performed to screen positive gene knockout mutants (Fig. S1). To generate the corresponding complementation strain of mutants, open reading frame of target gene plus its native promoter were amplified and fused with GFP tag, and the resulting amplicons, along with the geneticin-resistance gene were transformed into the protoplasts of the corresponding mutant. Similar strategies were conducted to generate other strains expressing GFP- or mCherry-fusion constructs. The primers used for genetic manipulation were presented in Table S2.

### Pathogenicity and DON production assays

Pathogenicity of tested strains was measured on wheat spikelets and wheat seedling leaves as described previously (67). Briefly, flowering wheat heads and 7-day-old wheat leaves were inoculated with tested strains and photographs were taken after inoculation at 25°C for 2 weeks and 1 week, respectively. DON detection kit (Wiseste Biotech Co. Ltd, China) was used to detect the DON production of tested strains after incubation in liquid TBI medium in the dark for 7 days. To measure the transcriptional abundance of the *TRI* genes, the total RNAs of tested strains were isolated by TRIzol. After the synthesis of ﬁrst-strand cDNA using the HiScript II 1st Strand cDNA Synthesis Kit (R212-02; Vazyme, China), quantitative real-time PCR (qRT-PCR) was conducted using ChamQ SYBR qPCR Master Mix (Q311-02, Vazyme). Actin was used as a reference gene. The primers used for qRT-PCR were also presented in Table S2.

### Western blotting

The fresh hyphae of tested strains were collected, and the proteins were isolated using protein lysis buffer [50 mM Tris-HCl (pH 7.5), 150 mM NaCl, 5 mM EDTA, and 1% Triton X-100]. After quantification, equal amounts of protein from different samples were loaded onto the 12% SDS-PAGE gel. After electrophoresis and membrane transfer, anti-GFP (ab32146; Abcam, Cambridge, UK), anti-mCherry (ab125096, Abcam), anti-His antibody (ab18184, Abcam), anti-H3 (no. 61475; Active Motif, La Hulpe, Belgium), anti-H3K18ac (no. 39756, Active Motif), anti-GST (M0807; Hua'an, Hangzhou, China), anti-GAPDH antibody (EM1101, Hua'an), and anti-Ub (ET1609-21, Hua'an) antibodies were used for immunoblot analyses.

### Pull-down and deubiquitination assays

Full-length cDNAs of *UBP14* and *UBI4* were amplified and cloned into pGEX-4T-1 and pET28A to generate GST-Ubp14 and His-Ubi4 constructs, respectively. The resulting vectors were then transformed into *Escherichia coli* strain BL21. After purification of recombinant proteins, purified GST or GST-Ubp14 was incubated with purified His-Ubi4. The mixtures were then incubated with Glutathione Sepharose for 4 hours and then washed 10 times with protein lysis buffer. Input and pulled proteins were subjected to SDS-PAGE gel and detected by immunoblot analysis. The interaction between Gcn5-Tom1 and Gcn5-Ubp14 was confirmed by a modified pull-down assay as described previously (33). For the Gcn5-Tom1 interaction, recombinant GST-Gcn5 protein purified from *E. coli* was incubated with total mycelial lysate of PH-1::Tom1-GFP-expressing *F. graminearum*. After incubation at 4°C for 4 hours, anti-GFP agarose beads (ChromoTek, Martinsried, Germany) were added to the mixture and incubated for another 2 hours. Then, the immunocomplexes were washed six times with washing buffer (50 mM Tris-HCl pH 8.0, 150 mM NaCl, 1% NP40). Bound proteins were eluted with sample loading buffer (FD002; Fudebio-tech, Hangzhou, China) and subjected to immunoblot analysis with an anti-GST antibody. For the interaction between Gcn5 and Ubp14, recombinant GST-Ubp14 protein purified from *E. coli* was incubated with total mycelial lysate of PH-1::Gcn5-GFP expressing *F. graminearum* at 4°C for 4 hours, then followed by a pull-down assay described above. The deubiquitination assays were conducted as described previously (18).

### Co-immunoprecipitation (Co-IP) and affinity capture-mass spectrometry analysis

Co-IP and affinity capture-mass spectrometry analysis were performed as described previously (68). Briefly, the total proteins of tested strains were extracted and incubated with the anti-GFP agarose. After washing with TBS (20 mM Tris-HCl, 500 mM NaCl, pH 7.5) for five times, proteins binding to the beads were detected with anti-mCherry antibody or analyzed by mass spectrometry. To test whether Ubp14 interacted with Gcn5 in the nucleus, cytoplasmic and nuclear fractions were prepared from the strains bearing Ubp14-GFP and Gcn5-mCherry using the strategies described previously (33). Then, anti-GFP agarose beads were added to the nuclear and cytoplasmic fractions. After incubation at 4°C for 4 hours, the beads were washed and the eluted proteins were subjected to immunoblot analysis with an anti-mCherry antibody. Detections with the anti-GAPDH and anti-histone H3 antibodies were used to distinguish cytoplasmic and nuclear proteins, respectively.

### Microscopic examinations

Fluorescent signals were detected using the Zeiss LSM780 confocal microscopy (Carl Zeiss AG, Germany). DAPI (4′,6-diamidino-2-phenylindole) of 10 µg/mL was used to stain the nucleus. Wheat glumes were inoculated with fresh mycelia of tested strains at 25°C for 48 hours before fixation in 2% glutaraldehyde, and the penetration structures were observed under Hitachi S-4700 field emission scanning electron microscope (Hitachi, Tokyo, Japan). Fluorescence intensity was quantified with the ImageJ software.

### Chromatin immunoprecipitation–quantitative PCR (ChIP-qPCR) analyses

ChIp-qPCR assays were performed as previously described (69). Briefly, the hyphae of the wild-type strain and Δ*ubp14* were collected after incubation in TBI medium for 48 hours. Fresh mycelia of each sample were ﬁxed with 1% formaldehyde for 10 minutes and then the reaction was stopped by glycine at a final concentration of 125 mM for 5 minutes. The cross-linked tissues were ground into fine powder in liquid nitrogen and then resuspended in a lysis buffer with protease inhibitor (Sangon, Shanghai, China). DNA was sheared into 200–500-bp fragments using Bioruptor Plus (UCD-300, Diagenode). The ChIP reaction was carried out with an anti-H3K18ac antibody (no. 39756, Active Motif). Immunoprecipitated DNA was enriched using protein A agarose beads (sc-2001; Santa Cruz Biotechnology, Santa Cruz, CA, USA) and washed sequentially with low-salt wash buffer, high-salt wash buffer, LiCl wash buffer, and Tris-EDTA (TE) buffer (69). Freshly prepared elution buffer (1% SDS, 0.1 M NaHCO_3_) was used to retrieve protein–DNA complex. The ChIP-enriched DNA was puriﬁed and quantiﬁed by qRT-PCR. The primers used for ChIp-qPCR assays were listed in Table S2.

## Data Availability

The data sets supporting the conclusions of this article are included within the article (and its supplemental material).

## SUPPLEMENTAL MATERIAL

Fig. S1 mbio.01499-23-s0001.tif Targeted gene deletion in *F. graminearum*. (A) Targeted genes and *HPH*/*NTC* genes are marked with large green and blue arrows, respectively. PCR primers were marked with small arrows. (B) Deletion mutants were identified by PCR assays. The sizes of the PCR products were indicated. (C) The DNA marker used in this S1 (B).

Fig. S2 mbio.01499-23-s0002.tif Mass spectrometry of the peptides containing K124 (A), K154 (B), K249 (C), K252 (D), K324 (E), and K335 (F) ubiquitination sites in Gcn5 protein.

Fig. S3 mbio.01499-23-s0003.tif The K252 in Gcn5 is important for fungal virulence and DON production. (A) Flowering wheat heads were inoculated with mycelial plugs from the indicated strains and the number of diseased spikelets per wheat head was measured at 14 dpi (B). (C) Mycelial plugs of the indicated strains were used to inoculate 7-day-old wheat seedlings and the lesion lengths were examined at 7 dpi (D). (E) Bar charts showing DON production in 7-day-old TBI cultures of the tested strains. Error bars indicate the standard deviation from three independent experiments. Statistical analysis was performed by Student's *t*-test. **P* < 0.05, ***P* < 0.01.

Fig. S4 mbio.01499-23-s0004.tif Ubp14 interacts with Gcn5 in the nucleus. Western blot showing the interaction between Ubp14 and Gcn5 in a coimmunoprecipitation assay. Cytoplasmic (left panel) and nuclear (right panel) fractions from the strains bearing Ubp14-GFP and/or Gcn5-mCherry (input) and the proteins eluted from the anti-GFP beads (elution) were detected using anti-GFP and anti-mCherry antibodies, respectively. Detections with the anti-GAPDH and anti-histone H3 antibodies were used to distinguish cytoplasmic and nuclear proteins, respectively.

Fig. S5 mbio.01499-23-s0005.tif The deletion of Ubp14 results in decreased H3K18ac enrichment at the promoters of *TRI* genes. (A) Chromatin immunoprecipitation-quantitative PCR (ChIP-qPCR) assays showing the enrichment of H3K18ac at the promoters of the *TRI4* (A), *TRI5* (B), and *TRI6* (C) in the indicated strains. Statistical analysis was performed by Student's *t*-test. **P* < 0.05, ***P* < 0.01, ****P* < 0.001.

Fig. S6 mbio.01499-23-s0006.tif The CRL4^Cdt2^ E3 ligase complex is dispensable for the Gcn5 ubiquitination. (A) Identification of putative CRL4^Cdt2^ E3 ligase complex in *F. graminearum*. (B) Western blot analysis of Gcn5 ubiquitination in the indicated strains expressing Gcn5-GFP. Gcn5-GFP was isolated and then immunoprecipitated with anti-GFP agarose beads from solubilized proteins. Ubiquitinated and basal Gcn5 proteins were detected by anti-ubiquitin and anti-GFP antibodies, respectively.

Table S1 mbio.01499-23-s0007.xlsx Putative Gcn5-interacting proteins identified in this study.

Table S2 mbio.01499-23-s0008.xlsx Primers used in this study.

ASM does not own the copyrights to Supplemental Material that may be linked to, or accessed through, an article. The authors have granted ASM a non-exclusive, world-wide license to publish the Supplemental Material files. Please contact the corresponding author directly for reuse.
