# Supplementary figures and images for: Ubiquitination of acetyltransferase Gcn5 contributes to fungal virulence in Fusarium graminearum
Source: mBio. 2023 Jul 28;14(4):e01499-23. doi: 10.1128/mbio.01499-23 (PMC10470610; doi:10.1128/mbio.01499-23)

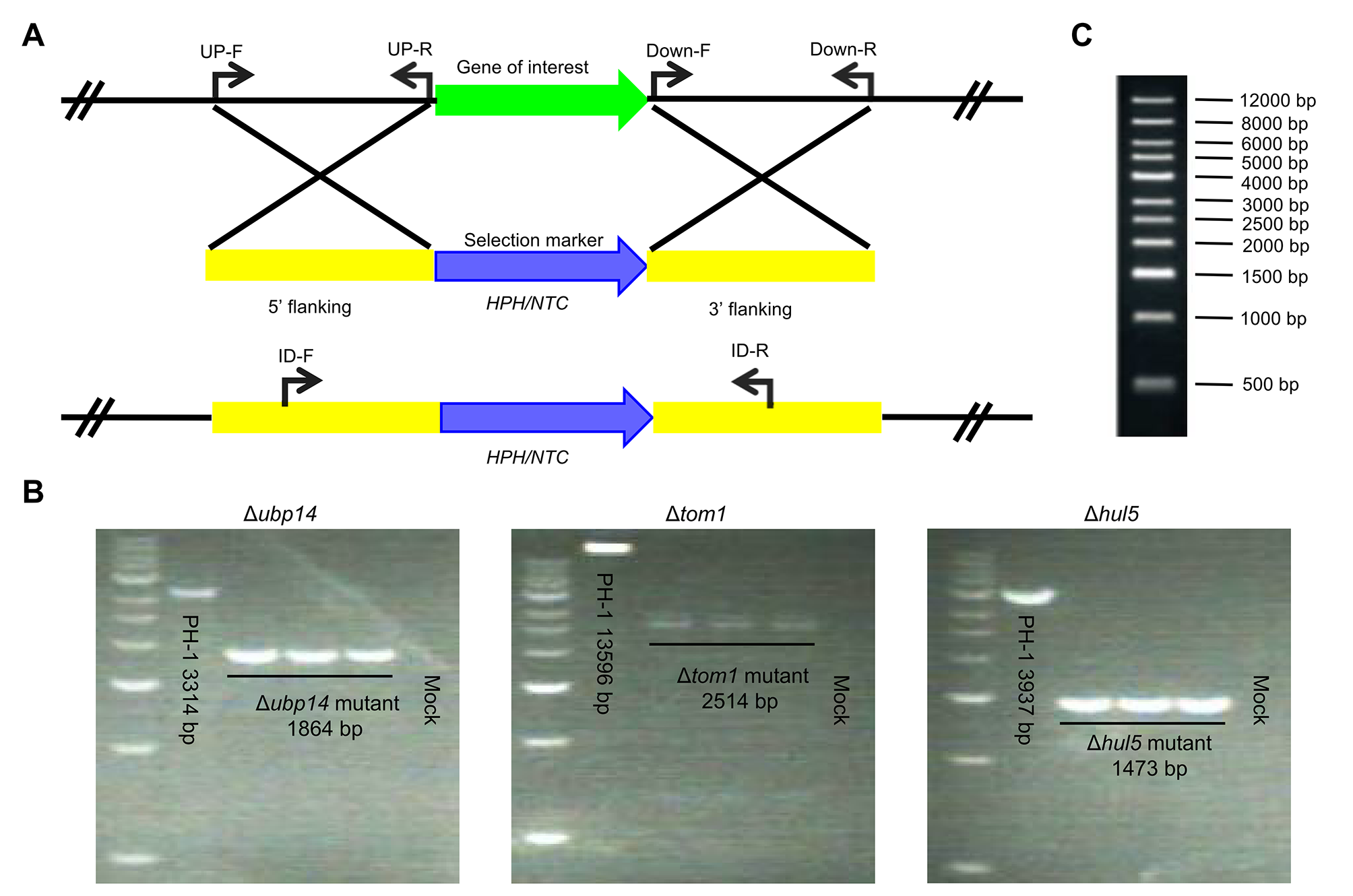

Supplement: Fig. S1 — Targeted gene deletion in F. graminearum. (A) Targeted genes and HPH/NTC genes are marked with large green and blue arrows, respectively. PCR primers were marked with small arrows. (B) Deletion mutants were identified by PCR assays. The sizes of the PCR products were indicated. (C) The DNA marker used in this S1 (B). [file mbio.01499-23-s0001.tif]

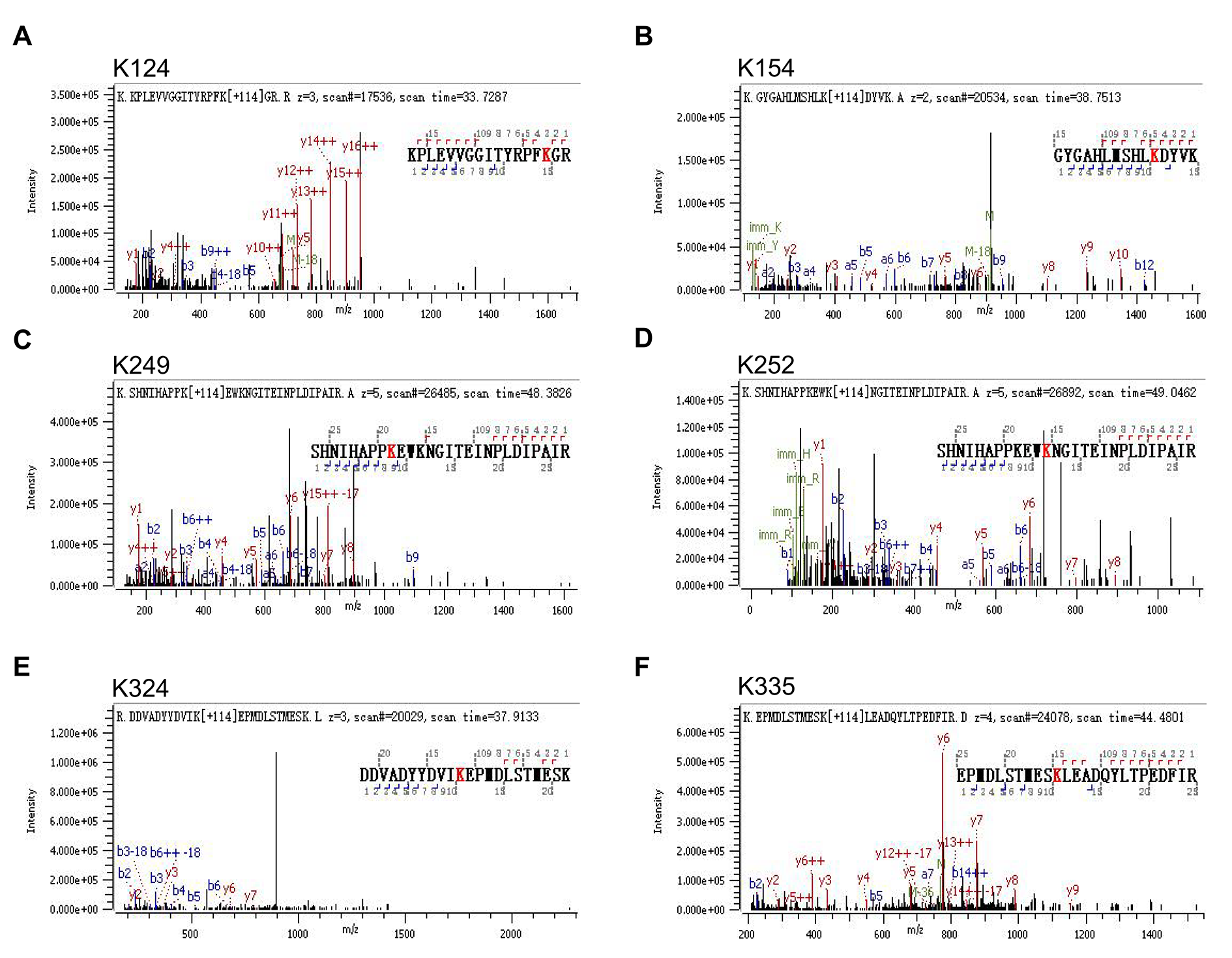

Supplement: Fig. S2 — Mass spectrometry of the peptides containing K124 (A), K154 (B), K249 (C), K252 (D), K324 (E), and K335 (F) ubiquitination sites in Gcn5 protein. [file mbio.01499-23-s0002.tif]

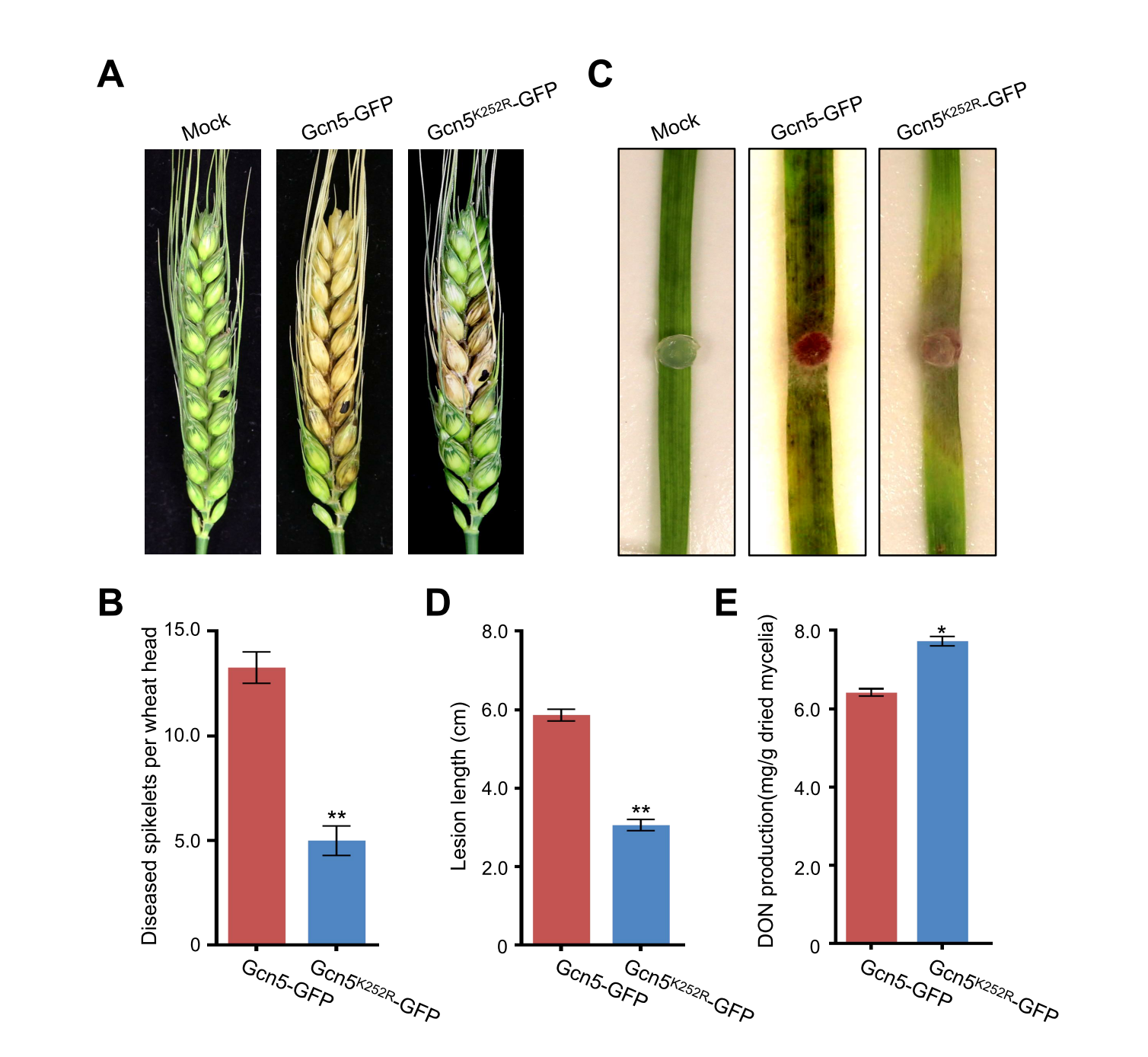

Supplement: Fig. S3 — The K252 in Gcn5 is important for fungal virulence and DON production. (A) Flowering wheat heads were inoculated with mycelial plugs from the indicated strains and the number of diseased spikelets per wheat head was measured at 14 dpi (B). (C) Mycelial plugs of the indicated strains were used to inoculate 7-day-old wheat seedlings and the lesion lengths were examined at 7 dpi (D). (E) Bar charts showing DON production in 7-day-old TBI cultures of the tested strains. Error bars indicate the standard deviation from three independent experiments. Statistical analysis was performed by Student's t-test. *P < 0.05, **P < 0.01. [file mbio.01499-23-s0003.tif]

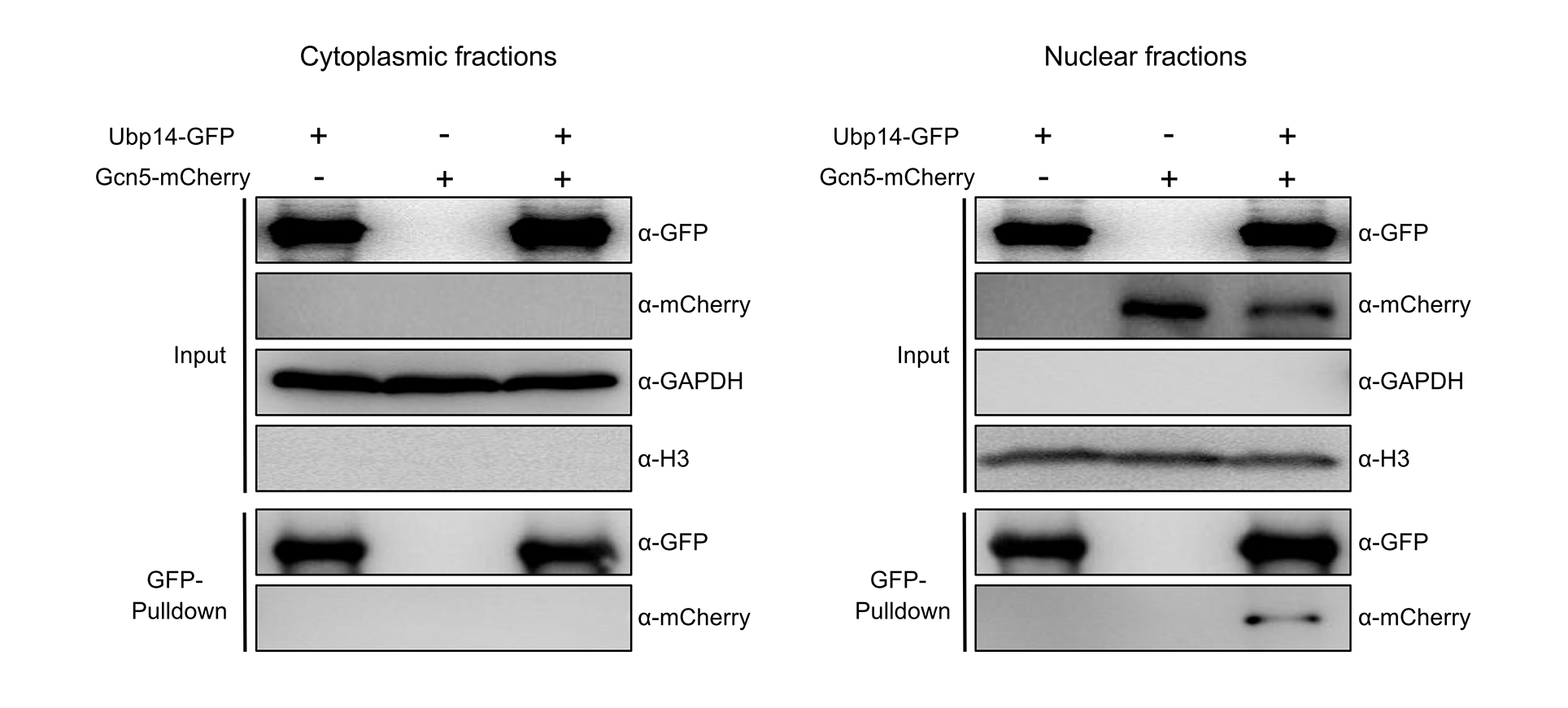

Supplement: Fig. S4 — Ubp14 interacts with Gcn5 in the nucleus. Western blot showing the interaction between Ubp14 and Gcn5 in a coimmunoprecipitation assay. Cytoplasmic (left panel) and nuclear (right panel) fractions from the strains bearing Ubp14-GFP and/or Gcn5-mCherry (input) and the proteins eluted from the anti-GFP beads (elution) were detected using anti-GFP and anti-mCherry antibodies, respectively. Detections with the anti-GAPDH and anti-histone H3 antibodies were used to distinguish cytoplasmic and nuclear proteins, respectively. [file mbio.01499-23-s0004.tif]

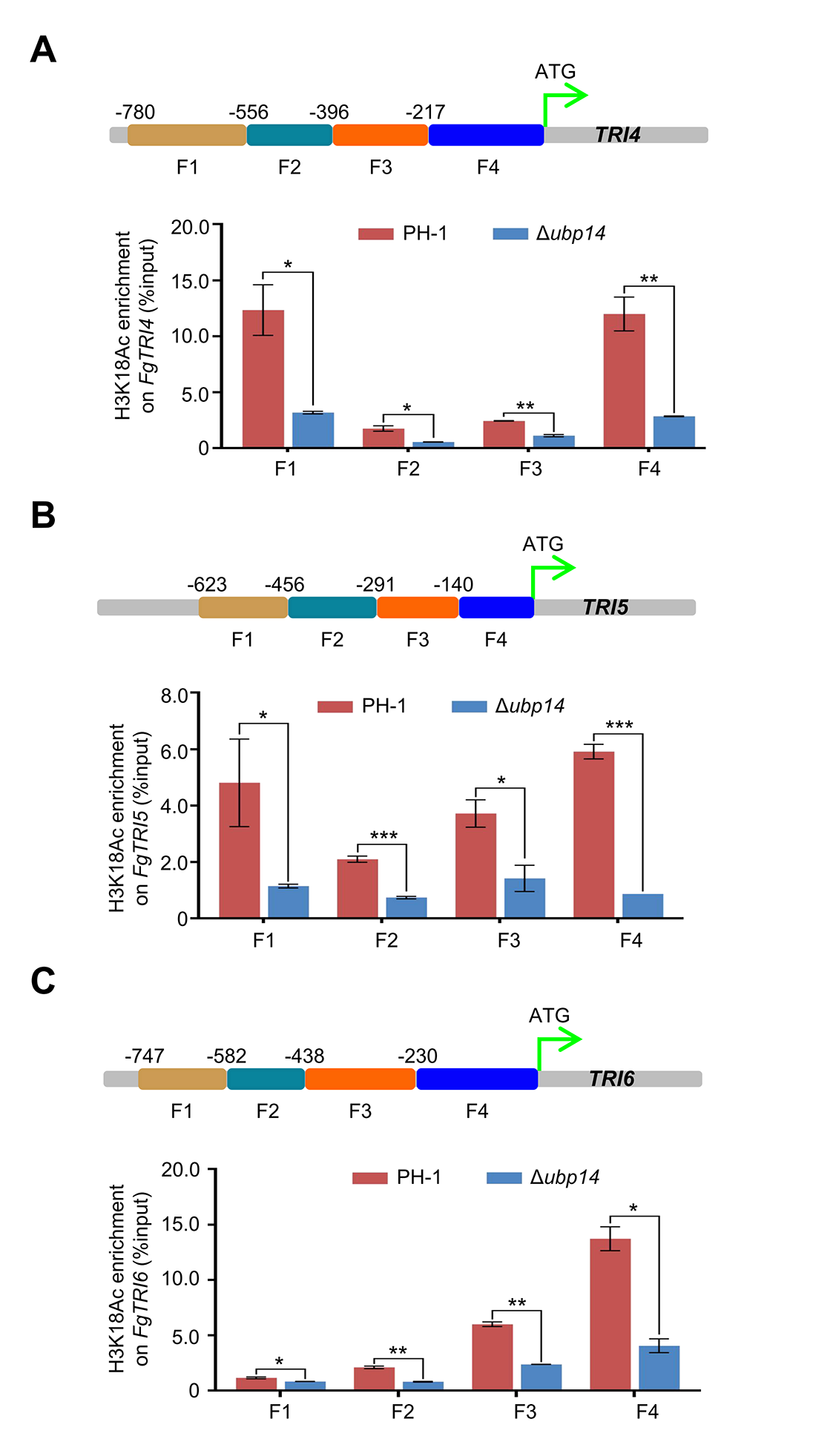

Supplement: Fig. S5 — The deletion of Ubp14 results in decreased H3K18ac enrichment at the promoters of TRI genes. (A) Chromatin immunoprecipitation-quantitative PCR (ChIP-qPCR) assays showing the enrichment of H3K18ac at the promoters of the TRI4 (A), TRI5 (B), and TRI6 (C) in the indicated strains. Statistical analysis was performed by Student's t-test. *P < 0.05, **P < 0.01, ***P < 0.001. [file mbio.01499-23-s0005.tif]

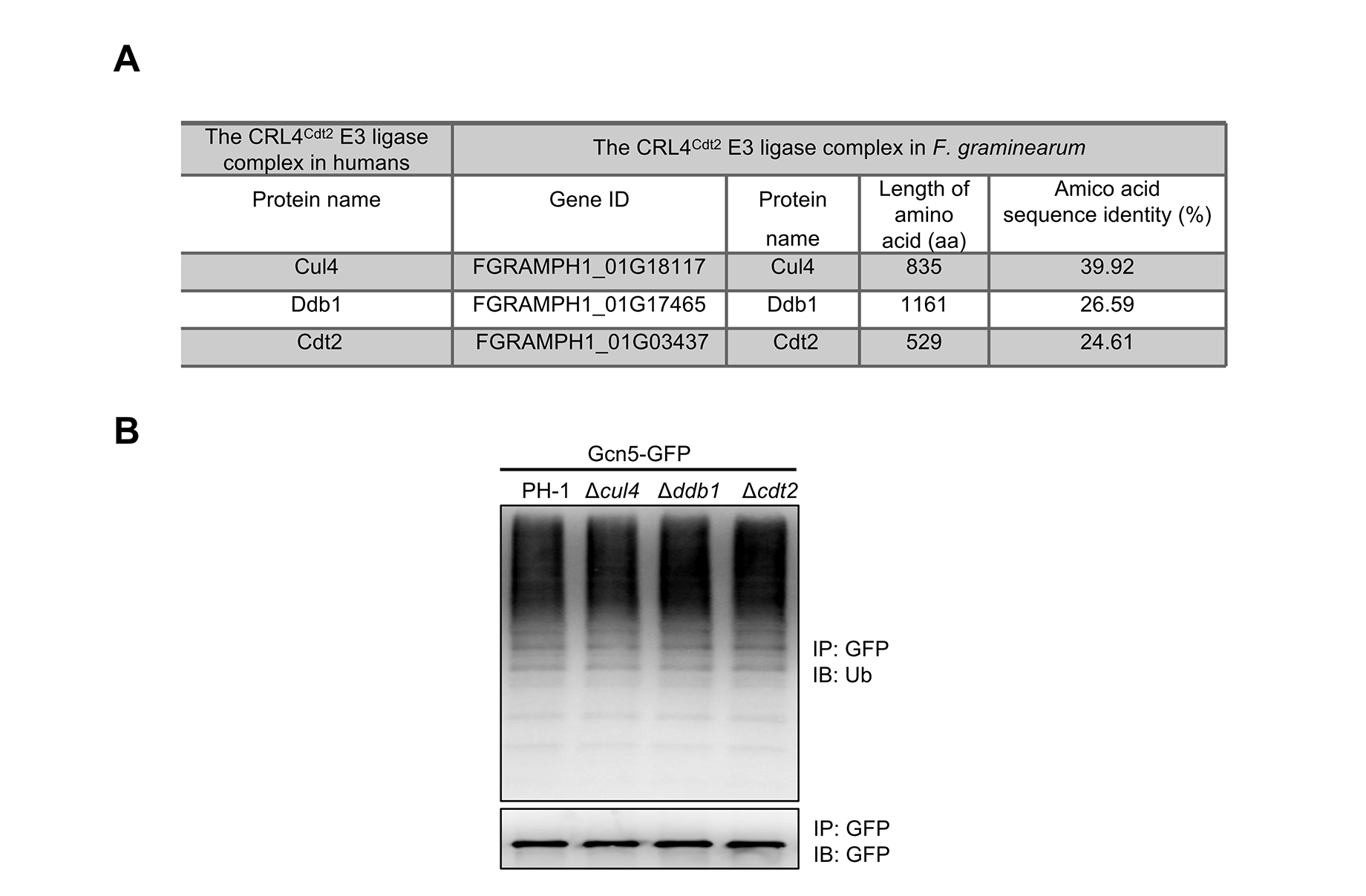

Supplement: Fig. S6 — The CRL4Cdt2 E3 ligase complex is dispensable for the Gcn5 ubiquitination. (A) Identification of putative CRL4Cdt2 E3 ligase complex in F. graminearum. (B) Western blot analysis of Gcn5 ubiquitination in the indicated strains expressing Gcn5-GFP. Gcn5-GFP was isolated and then immunoprecipitated with anti-GFP agarose beads from solubilized proteins. Ubiquitinated and basal Gcn5 proteins were detected by anti-ubiquitin and anti-GFP antibodies, respectively. [file mbio.01499-23-s0006.tif]
